# Analog Control of Reconfigurable GHz Resonances from Chiral Spin Texture Ensembles

**DOI:** 10.1002/adma.202521980

**Published:** 2026-03-04

**Authors:** T. S. Suraj, Jifei Huang, Hui Ru Tan, Jing Zhou, Abhijit Ghosh, Hang Khume Tan, May Inn Sim, Alexander K. J. Toh, Xiaoye Chen, Anjan Soumyanarayanan

**Affiliations:** ^1^ Department of Physics National University of Singapore Singapore Singapore; ^2^ Institute of Materials Research and Engineering, Agency for Science, Technology and Research Singapore Singapore

**Keywords:** magnonics, micromagnetics, microwave spectroscopy, spin textures, spintronics

## Abstract

Gigahertz excitations of magnetic films are widely explored for energy‐efficient, high‐frequency microelectronics. The advent of nanoscale chiral spin textures (CSTs) with topological dynamics promises novel resonance characteristics. However, prior works on technologically relevant chiral multilayers encountered key material constraints, precluding the realization of functional CST resonances. We address this by engineering a minimally damped, strongly chiral multilayer with a robust broadband resonance spectrum. Microwave spectroscopy, Lorentz microscopy, and simulations elucidate contrasting resonance features on either side of zero magnetic field arising from distinct irreversible CST transitions. A simple analytical model can quantitatively describe these robust inter‐textural resonances over the entire field‐frequency range. Crucially, in situ CST reconfigurability enables analog tunability of the resonant dispersion ‐ with wide‐band, deterministic, non‐linear (or linear) modulation via the input knob. Our work unlocks the microwave potential of multilayer CSTs by leveraging their unique thermodynamics. It opens the door to fabrication‐free reconfigurable magnonics, toward broadband transmission and unconventional computing.

## Graphical abstract

Tunable microwave components ‐ essential for wireless technology ‐ typically require costly, customized nanofabrication. We show that ambient spin texture ensembles (e.g., magnetic skyrmions) in chiral multilayer films can enable reconfigurable microwave resonators. By controlling magnetic field history, we achieve widely programmable GHz dispersion tuning without physical nanopatterning. This materials‐driven approach enables compact, energy‐efficient devices for next‐generation communications and unconventional computing.

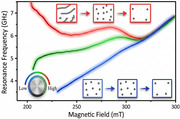

## 1 Introduction

Modern infocomm technology increasingly demands robust interfaces for computing and communications, requiring devices operating efficiently at GHz frequencies [1]. Magnonics – a promising high‐frequency avenue – employs collective spin excitations (“magnons”) in CMOS‐compatible films for processing and transmitting information [1, 2, 3]. Magnon generation, transmission, and detection is typically achieved by periodic nanomagnetic configurations known as magnonic crystals (MCs) [3, 4]. State‐of‐the‐art MCs use nanopatterned magnetic media to precisely modulate nanoscale magnetization to achieve desired spin‐wave dispersion characteristics [4, 5, 6, 7]. Therefore, realizing in situ, reconfigurable spin wave dispersion has tremendous potential for beyond‐CMOS applications, including data processing, computing, and variable‐bandwidth communications [1, 2, 3, 8].

The emergence of room‐temperature (RT) chiral spin textures (CSTs) in multilayer films has paved a new scientific frontier [9, 10], with considerable relevance to magnonics [11, 12]. Whirling arrangements of spins amidst a uniform background, CSTs present as ensembles of round skyrmions and/or elongated stripes [13, 14, 15, 16]. The nanoscale size, topological protection, and electrical pliability of skyrmions are promising as candidate mobile bits [9, 10]. Meanwhile, the distinctive spatiotemporal attributes of CSTs manifest as a rich spectrum of localized and extended GHz resonances, in contrast to uniform precession in conventional ferromagnets (FMs) [17, 18, 19]. Notably, their tunability with fields and currents could enable bottom‐up MCs with in situ programmability, with lucrative technological prospects [11, 12, 20, 21, 22].

Several works have investigated microwave resonances of CSTs, primarily within bulk crystalline helimagnets that host fixed‐wavelength noncollinear phases at cryogenic temperatures [11, 19]. The microwave spectrum of these isotropic helimagnets features localized resonances of fixed skyrmionic lattices and confined excitations from related 1D spirals over fixed fields and frequencies [17, 18, 23, 24], with intriguing functional characteristics [25, 26]. Recently, efforts on technologically relevant chiral multilayer films have shown hints of analogous individual and collective CST resonances [27, 28, 29]. However, multilayer works have been limited to large (∼20) stacked repetitions or low chirality, yielding broadened resonances over a small operating range, with limited resolution and complex subtleties [27, 28, 29, 30]. Chiral multilayers are yet to offer novel resonant functionalities – a critical bottleneck to employing CSTs for magnonics [11, 12].

Here, we show robust CST resonances over a broad field‐frequency range on minimally damped, strongly chiral Ir/Fe/Co/Pt multilayers. In contrast to literature, here the interplay of anisotropy and chirality yields distinct resonance features on either side of zero magnetic field (ZF), arising from irreversible spin‐textural transitions. Crucially, across the entire range, the spectral dispersion has a direct, analytical relationship with the underlying CST configuration. We employ the deterministic response and analog tunability of CSTs as knobs to demonstrate the wide‐ranging programmability of their resonant microwave dispersion, with direct implications for reconfigurable magnonics.

Crucially, across the entire range, the spectral dispersion has a direct, analytical relationship with the underlying CST configuration. We employ the deterministic response and analog tunability of CSTs as knobs to demonstrate the wide‐ranging programmability of their resonant microwave dispersion, with direct implications for reconfigurable magnonics.

## 2 Chiral Spin Textures

**FIGURE 1:**
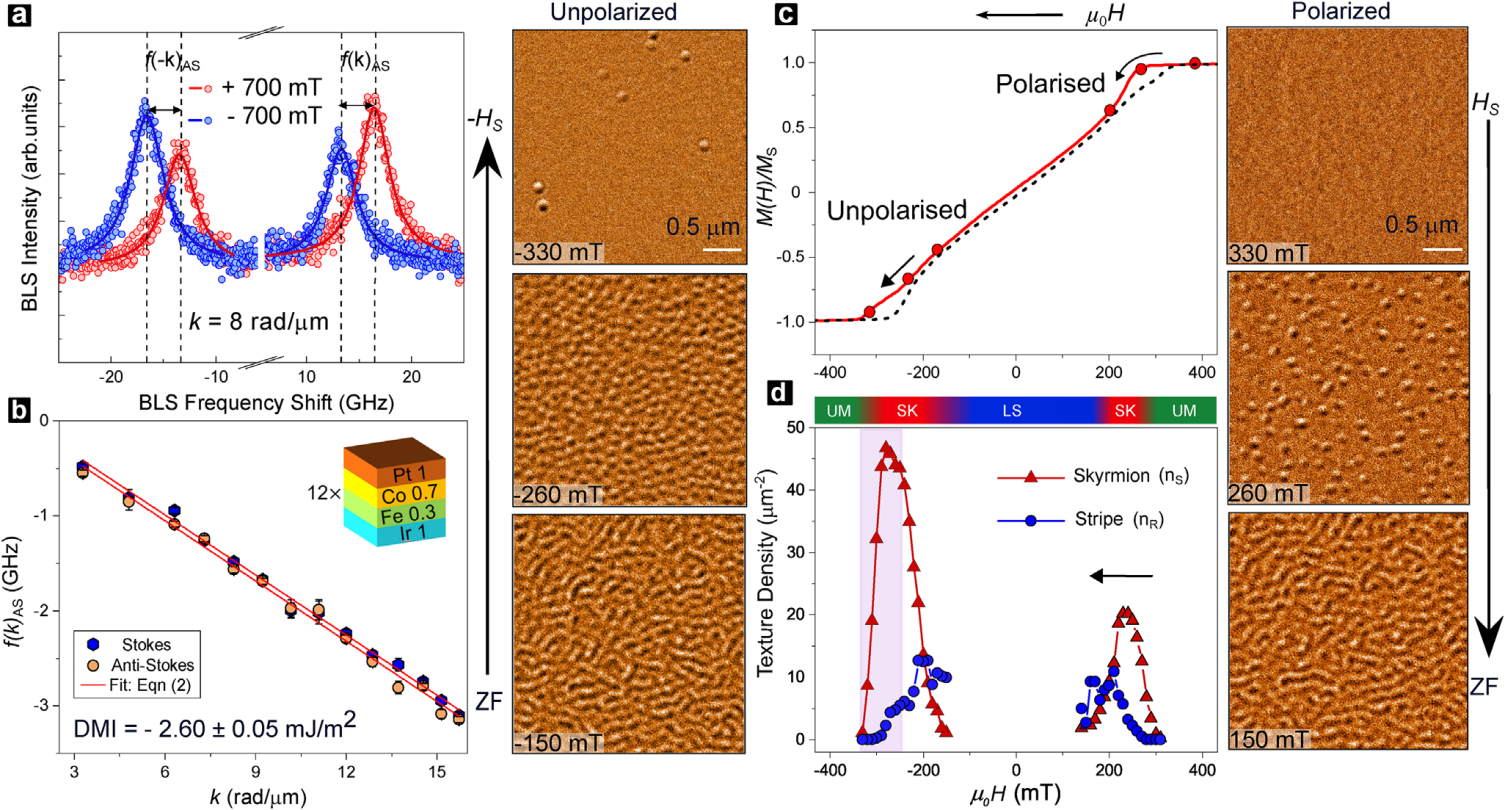
Stack Properties and Spin Texture Evolution. (a) Representative BLS spectra at in‐plane (IP) magnetic field, $\mu_{0}H_{\mathrm{ext}}=\pm 300$ mT. Dashed lines identify frequency shift $f{\left(\pm k\right)}_{\mathrm{AS}}$ between the Stokes and anti‐Stokes peaks. (b) Plot of frequency shift $f{\left(k\right)}_{\mathrm{AS}}$ against incident wavevector k for both peaks, and linear fits (lines). Inset: Ir/Fe/Co/Pt multilayer stack structure (layer thicknesses in nm). (c) Normalized out‐of‐plane (OP) magnetization hysteresis loop, $M(H)/M_{S}$ (arrow indicates sweep direction, $M_{S}$: saturation magnetization). Red circles indicate field‐points for Lorentz TEM (LTEM) images (left, right insets) for field sweeps on field‐polarized ($+H_{S}$ to zero, right: red arrow) and unpolarized (zero to $-H_{S}$, left: purple arrow) sides. (d) LTEM‐measured density of skyrmion ($n_{S}$) and stripe textures ($n_{R}$) with varying field (see Experimental Section). Shaded pink box highlights hysteretic region (focus of this work). Top inset: color scalebar shows statistically determined magnetic phases – uniform magnetization (UM), labyrinthine stripe (LS), skyrmion (SK).

To host and detect robust microwave resonances, the multilayer stack must have strong chirality to stabilize nanoscale CSTs, and reduced damping and broadening effects for coherent response [11, 12]. We utilize the Ir/Fe/Co/Pt platform that hosts smoothly tunable Néel CSTs [16, 31]. The chosen stack composition, [Ir(1)/Fe(0.3)/Co(0.7)/Pt(1)]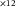 (thicknesses in nm, see Figure 1b inset), is expected to host dense CST configurations with optimal signal‐to‐noise for imaging and spectroscopy. Wave‐vector‐resolved Brillouin light scattering (BLS) spectra (Figure 1a) show expected asymmetry in spin wave dispersion arising from the interfacial Dzyaloshinskii‐Moriya interaction (iDMI). The measured iDMI, D≃−2.60±0.05 mJ m^−2^ (Figure 1b), is large compared with literature [32], and consistent with the atomically sharp interfaces in our stacks [33]. Following established conventions [34], its negative sign indicates that ensuing spin textures would have left‐handed Néel chirality. Meanwhile, the magnetization hysteresis loop, M(H), is characteristically sheared (see Figure 1c), with moderate effective anisotropy ($K_{\mathrm{eff}}≃0.1$ MJ $m^{-3}$).

The chiral stability parameter, $\kappa =\pi D/4\sqrt{AK_{\mathrm{eff}}}$, governs the prevalence of CSTs, where A is the exchange stiffness. For our stack, κ≃1.5 is well above unity (Section S1), suggesting the presence of dense CST ensembles. Indeed, LTEM experiments (Figure 1c:insets) evidence nanoscale skyrmions and stripes with fixed Néel chirality (Section S2), that should manifest uniformly through the stack thickness (Section S5) [31]. As H is swept down from positive saturation ($+H_{\max}\to 0$, Figure 1c: right), defined as the field‐polarized side, sparse isolated skyrmions (∼50nm size) nucleate at $∼0.8H_{S}$, and rapidly elongate into stripes, forming a labyrinthine (LS) state at zero field (ZF). As H is swept from ZF towards negative saturation ($0\to -H_{\max}$, Figure 1c: left), termed the field‐unpolarized side, the LS transforms into discrete stripes, which fission into a dense, disordered skyrmion lattice [35, 36]. Finally, these skyrmions annihilate to the uniform state at $-H_{S}$. The distinct textural evolution mechanisms for polarized and unpolarized sides yield asymmetric densities of skyrmions, $n_{S}\left(H\right)$ (see Figure 1d).

## 3 Resonance Modes Overview

**FIGURE 2:**
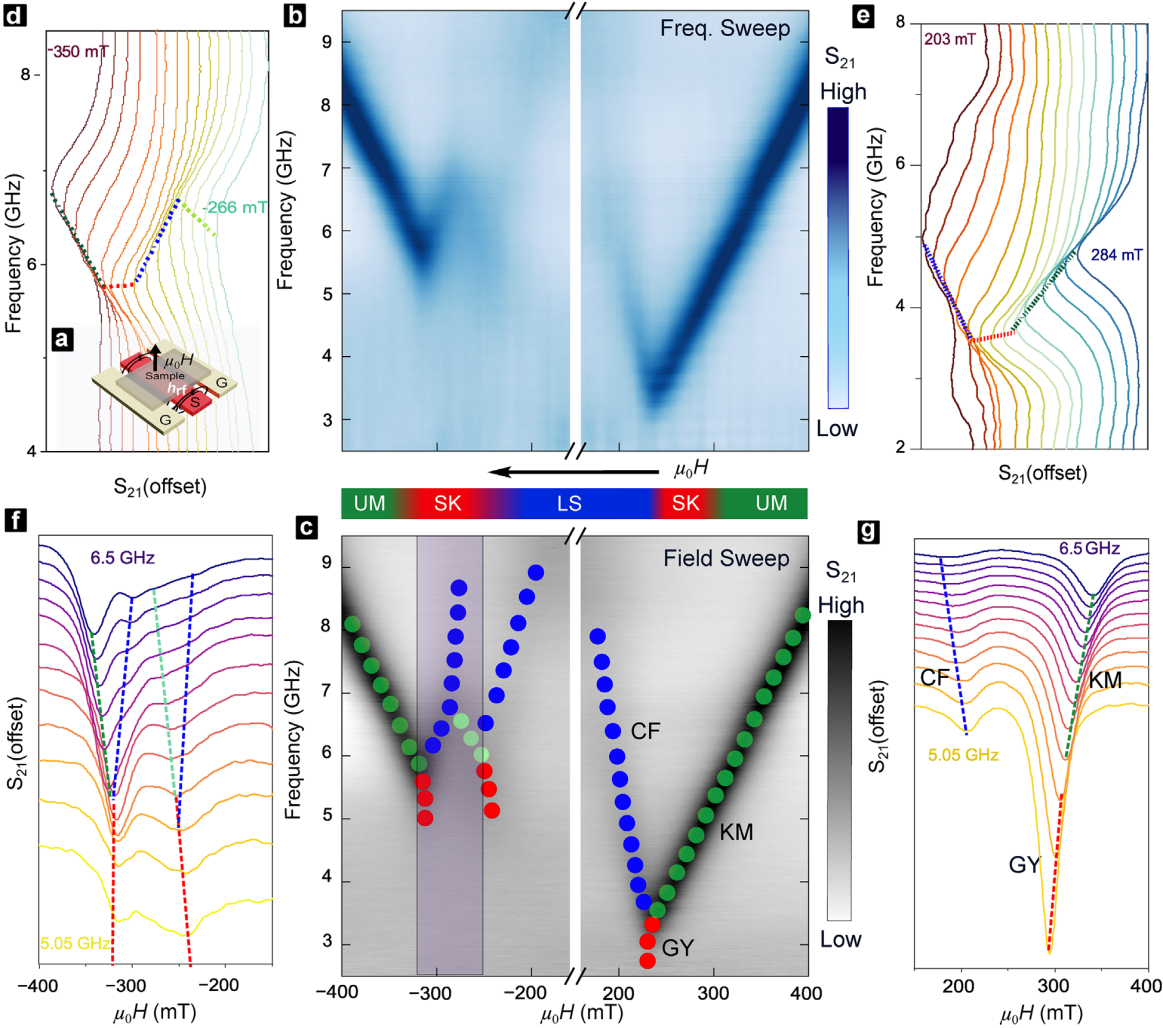
Frequency‐ and Field‐Dependent Microwave Absorption Spectroscopy (MAS). (a) Schematic of MAS setup. Coplanar waveguide measures RF transmission ($S_{21}$, black) of the sample from IP excitation field ($h_{\mathrm{rf}}$) under static OP field (H). (b‐c) MAS color plots of normalized $S_{21}$ against frequency (f), and OP field ($\mu_{0}H$), using (b) frequency‐sweep (constant‐H) and (c) field sweep (constant‐f) protocols. Distinct resonance modes are annotated in (c): Kittel mode (KM: green), gyrotropic skyrmion mode (GY: red) and confinement mode (CF: blue). Purple box highlights hysteretic region, arrow indicates field sweep direction, centre inset (color scalebar) indicates LTEM‐imaged evolution of magnetization states (from Figure 1d). (d)/(f) and (e)/(g) insets show representative linecut spectra for unpolarized and polarized sides respectively of (b)/(c), taken at selected fields (frequencies) for both sweep protocols. Solid lines indicate identified resonance modes.

Broadband MAS experiments were conducted at room temperature to characterize CST resonances using a home‐built coplanar waveguide (CPW) in flip‐chip geometry (Figure 2a). Transmission spectra ($S_{21}$) were acquired over frequencies up to 26 GHz, with the out‐of‐plane field ($\mu_{0}H$) swept across ±750 mT (see Experimental Section). Above saturation, uniform magnetization precession (Kittel mode) [37] produces sharp resonances with linewidths corresponding to an effective damping $\alpha_{\mathrm{eff}}∼0.02$ [38], among the lowest reported for chiral multilayers [11, 28, 29, 30] (Section S1). Next, high‐resolution spectra were acquired using frequency‐sweep (Figure 2b, (d)&(e): constant‐H linecuts) and field‐sweep (Figure 2c, (f)&(g): constant‐f linecuts) protocols to study the resonant modes in different textural phases (Figure 2c, colored markers). Spectral color plots $S_{21}\left(f,H\right)$ from both methods (Figure 2b,c) show quantitatively consistent resonances, confirming their robustness and ruling out protocol‐ or setup‐ induced artifacts. This consistency allows clear identification of dispersing modes (Figure 2c, colored markers) and facilitates investigation of their microscopic origin (Figure 2c, top inset).

The MAS color plots show distinct resonance characteristics for field‐polarized (right) and unpolarized (left) sides. On the polarized side ($+H_{S}\to \mathrm{ZF}$, Figure 2b,c: right), the linear Kittel mode (KM) exhibits a reduced slope below $H_{S}$ and splits into two modes with positive and negative dispersion (df/dH), forming a ‘Y'‐shaped feature consistent with prior works on helimagnets and multilayers [18, 23, 24, 27, 28, 29]. The narrow‐field tail (GY mode) arises from gyrotropic, counter‐clockwise skyrmion rotation, and the wide‐field upturn (CF mode) from precession of confined regions between stripes [11, 17, 18, 19]. On the unpolarized side (ZF to $-H_{S}$, Figure 2: left), in addition to the near saturation KM, its GY tail, and the low‐field CF, we find three more resonances in the field‐region hosting hysteretic spin‐textural transitions (Figure 2b,c: purple). These new features form an upturn, downturn, and tail – visually similar to known resonances – collectively forming a “YY”‐shaped unpolarized spectrum. Meanwhile, the overall upward shift of the unpolarized spectrum is expected for anisotropic multilayers [28, 29]. Crucially, this unpolarized “YY” spectrum is absent in multilayers with reduced chirality (κ<1, Section S4), which exhibit “Y”‐shaped dispersions on both sides, consistent with prior reports on such samples  [27, 28, 30, 39]. This unique “YY” resonant characteristic of κ>1 multilayers is evidenced by detailed MAS measurements across five distinct κ values (Section S4). We note that the κ<1 and κ>1 samples exhibit comparable interfacial quality across static and dynamic magnetic characterization (Section S1). This indicates that the observed dispersion differences originate from variations in intrinsic magnetic properties, rather than interface quality related extrinsic effects.

**FIGURE 3:**
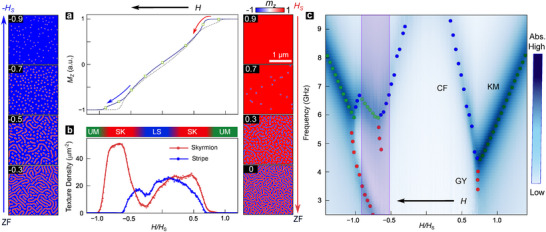
Simulated Spin Textures and Resonances. (a) Micromagnetically simulated hysteresis loop of normalized OP magnetization ($m_{z}$) for stack parameters emulating experiments (Experimental Section). Insets: representative $m_{z}$ images of CSTs for field‐polarized (right) and unpolarized (left) sides. (b) Field evolution of density of skyrmion ($n_{S}$: red) and stripe (blue) textures. (c) Simulated MAS color plot (Experimental Section) with varying f and $H/H_{S}$. Overlaid colored markers indicate distinct resonance modes (KM, GY, CF: c.f. Figure 2), purple box highlights hysteretic region.

We performed detailed micromagnetic simulations to study the spatiotemporal origin of these resonance modes using stack structures, magnetic parameters, and recipes equivalent to experiments (Section S5) [31]. Simulations reproduce a sheared hysteresis loop (Figure 3a), Néel spin‐textures, and asymmetric skyrmion‐stripe evolution with distinct texture densities on field‐polarized and unpolarized sides (Figure 3a,b), consistent with Figure 1d and our previous works [31, 36]. Magnetization dynamics were simulated by analyzing broadband response to an IP excitation field for varying H (Experimental Section). The simulated MAS plot (Figure 3c reproduces the “Y” and “YY” features on the field‐polarized and unpolarized sides respectively, as in experiments. Here, the low‐frequency GY mode persists over a larger frequency range in the absence of experimental signal‐to‐noise constraints. Meanwhile, the increased field separation of two unpolarized ‘Y'‐features can be attributed to the larger unpolarized “plateau” of skyrmion density (Figure 3b) relative to experiments (Figure 1d). The established overall consistency with experiments enables us to employ simulations to investigate the unpolarized hysteretic resonance modes.

## 4 Microscopic Origin of Resonance Modes

**FIGURE 4:**
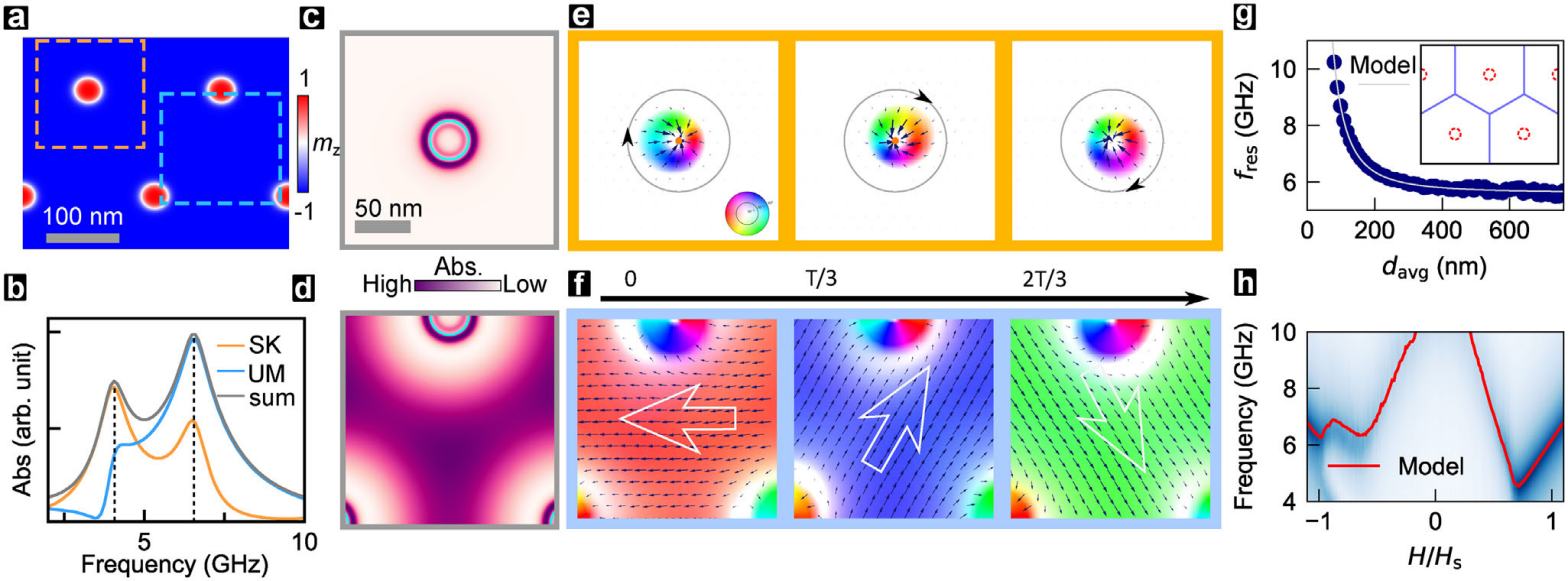
Simulated Origin of Resonance Modes. (a) Simulated $m_{z}$ image of skyrmion lattice ($\mu_{0}H=-200$ mT) used to elucidate resonance modes. Selected dashed square regions have skyrmion (SK: orange) and uniform (UM: cyan) characteristics. (b) MAS spectra for (a), over the full region, and for the SK‐like and UM‐like square regions (centrally Gaussian weighted). Dashed lines identify GY, CF resonance peaks. (c, d) Spatial absorption profile of (c) skyrmionic GY mode (f=4.1 GHz) and (d) inter‐skyrmion CF mode (f=6.8 GHz). Cyan lines represent skyrmion boundary. (e, f) Temporal magnetization snapshots for (e) f=4.1 GHz (GY‐like) and (f) f=6.8 GHz (CF‐like mode), at time, t=0, 1/3T, and 2/3T, for oscillation period T. Small arrows (orientation) and color scale (magnitude, log‐scale) denote the IP magnetization (1 % perturbation of primarily OP spin for CF mode). (g) Variation of resonance frequency, $f_{\mathrm{res}}$, of CF mode with inter‐skyrmion distance ($d_{\mathrm{avg}}$, see Experimental Section). Grey line shows an analytical fit using the toy model (see text). Inset image shows medial axes (blue lines) of a skyrmion lattice. (h) Simulated MAS (H,f) color plot (Figure 3c), with overlaid resonance modes predicted by toy model (red line).

To establish their origin, we study the spatial MAS variation for a small, close‐packed skyrmion array (Figure 4a, $\mu_{0}H=-200$ mT), and their temporal magnetization evolution at characteristic frequencies. Spatially resolved spectra (Figure 4b) reveal a low‐frequency mode arising from the skyrmionic region (Figure 4a: orange square), and a high‐frequency mode originating from inter‐skyrmion regions (Figure 4a: cyan square). The absorption profile of the low‐frequency resonance confirms its skyrmionic origin (Figure 4c) and prominence for boundary spins ($m_{z}=0$), while its temporal magnetization evolution shows counter‐clockwise gyration of the skyrmion core (Figure 4e). In contrast, analogous spatiotemporal analysis of the high‐frequency resonance (Figure 4d,f) shows that it arises from the uniform precession of background magnetization and is maximal in the region between skyrmions. The low spatial absorption in near‐skyrmion regions is attributed to strong local interactions ($\propto {\left(\nabla m\right)}^{2}$) [40], which effectively act as a boundary for resonating spins.

The inter‐texture resonances crisscrossing the f‐H spectral map motivate a quantitative description. Inspired by the 2D vibrating drumhead, we propose a phenomenological model (details in Experimental Section), wherein the resonance frequency $f_{\mathrm{res}}\left(H\right)$ is determined by the characteristic inter‐texture distance $d_{\mathrm{avg}}\left(H\right)$

(1) $$f_{\mathrm{res}}\left(H\right)=C/\left(d_{\mathrm{avg}}^{2}\left(H\right)\right)+f_{K}\left(H\right)$$

C, the only fit parameter, relates to exchange strength, and is obtained from the dispersion of the simulated skyrmion lattice (Figure 4g). Meanwhile, $f_{K}\left(H\right)$ is the well‐known linear dispersion of the uniformly precessing KM [37]. The resonant dispersion predicted by the toy model (Figure 4h: lines) exhibits excellent quantitative agreement with that of simulated textures (Figure 4h: plot), and thus also with experiments. Importantly, its ability to emulate the dispersions of Kittel‐like (df/dH>0) and CF modes (df/dH<0) via contrasting texture evolutions provides a valuable physical picture of the observed resonance spectrum (Figure 4h).

As the field is reduced from $+H_{S}$ (Figure 4h: right), nucleation of sparse skyrmions renormalizes the Kittel mode and leads to the emergence of GY and CF modes. While the GY mode disappears abruptly, the CF mode evolves predictably with increasing texture density, well into the labyrinthine stripe phase. Below ZF (Figure 4h: left), the CF mode disperses with stripe density, and pairs with a GY mode as elongated stripes fission into a dense lattice [36]. This topologically stabilized lattice maintains its density over a sizable field range (∼50 mT), over which precession of uniform background magnetization disperses as a quasi‐Kittel mode. Eventually, skyrmion density reduces with field, producing another CF mode, which pairs with a GY mode of isolated skyrmions to yield the Kittel mode at $-H_{S}$.

These results establish several key insights on microwave resonances of CSTs. First, any thermodynamic transitions of CSTs – formation, annihilation, or irreversible interconversion – should produce a “Y”‐shaped MAS feature. Second, the distinction in polarized and unpolarized MAS spectra arises from their contrasting CST evolution. In isotropic helimagnets and typical multilayers (κ<1), the skyrmion‐to‐stripe transition is reversible, and both polarized and unpolarized sides have one “Y”‐shaped resonance structure (Section S4), due to skyrmion formation and annihilation, respectively [18, 23, 24, 28]. For strongly chiral multilayers (κ>1), the unpolarized side has an additional irreversible transition – stripe‐to‐skyrmion fission [31, 35] – which produces another ‘Y'‐shaped resonance structure (Section S4), yielding an overall ‘Y'‐shape, in contrast to prior works.

## 5 Field Reconfigurability of Resonances

**FIGURE 5:**
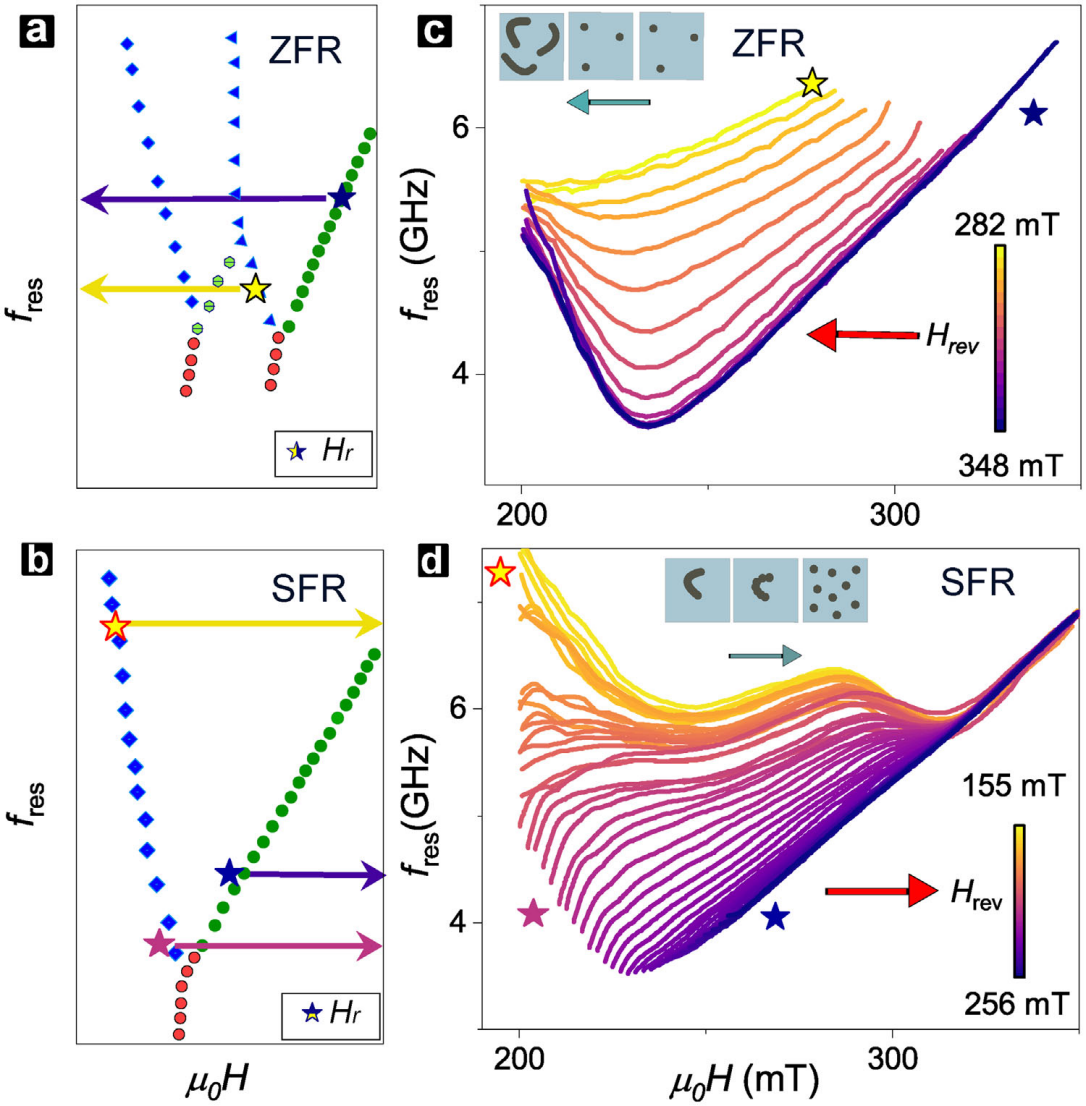
Field Reversal MAS Resonances. (a, b) Skeletal MAS resonance plots (from Figure 2c) to reference minor loop sweeps from reversal field $H_{\mathrm{rev}}$. The two sweep protocols are (a) field‐unpolarized zero field reversal (ZFR), and (b) field‐polarized saturated field reversal (SFR). Arrows indicate field‐sweep direction, coloured stars identify representative $H_{\mathrm{rev}}$. (c, d) Minor loop resonance spectra, $f_{\mathrm{res}}\left(H\right)$, with varying $H_{\mathrm{rev}}$ (color‐coded) for (c) ZFR and (d) SFR. Each $f_{\mathrm{res}}\left(H\right)$ spectrum is condensed from a series of f‐sweep spectra (Experimental Section). Insets: schematic texture evolution for (c) ZFR (skyrmions to stripes) and (d) SFR (stripe to skyrmion fission).

CST configurations in strongly chiral multilayers can be precisely tailored via field history, governed by the reversal field, $H_{\mathrm{rev}}$ [35, 36]. Inspired by established field‐reversal characterization techniques [33, 35, 41], we employed field‐reversal MAS to study the $H_{\mathrm{rev}}$ tunability of CST resonances. We adopted two distinct sweep protocols for the field‐unpolarized and field‐polarized sides. The zero‐field reversal (ZFR) protocol was initialized on the unpolarized side (Figure 5a, stars) by sweeping the field from $-H_{s}$ through zero field (ZF) to $+H_{\mathrm{rev}}$ (see Experimental Section). MAS spectra, $S_{21}\left(f\right)$, were recorded over the return sweep from $+H_{\mathrm{rev}}$ back to ZF (Figure 5a, arrows), yielding a 2D spectral plot similar to Figure 2b. The Resonant peak positions $f_{\mathrm{res}}\left(H\right)$ (Figure 5c,d) are obtained via Lorentzian fitting of MAS spectra (see Experimental Section). Conversely, the saturated‐field reversal (SFR) protocol was initialized on the field‐polarized side (Figure 5b) by sweeping the field from $+H_{s}$ to $+H_{\mathrm{rev}}$ (without passing through ZF). In this case, spectra were recorded during the return sweep ($+H_{\mathrm{rev}}\to +H_{s}$, Figure 5d).

Figure 5c,d illustrate the remarkable tunability of $f_{\mathrm{res}}\left(H\right)$ achieved over an identical field range, by varying sweep protocol and $H_{\mathrm{rev}}$. First, the two extreme ends, $H_{\mathrm{rev}}∼H_{S}$ for ZFR (Figure 5c: purple) and $H_{\mathrm{rev}}\ll H_{S}$ for SFR (Figure 5d: yellow) are expectedly similar to major loop resonances for Figure 5b (polarized) and Figure 5a (unpolarized) respectively, reaffirming the validity of the sweep protocols. For ZFR, as $H_{\mathrm{rev}}$ is reduced from $H_{S}$, $f_{\mathrm{res}}\left(H\right)$ qualitatively retains its “V”‐like shape, while the KM and CF modes gradually shift to higher frequencies and exhibit reduced slopes. Meanwhile, for SFR, $f_{\mathrm{res}}\left(H\right)$ is linear for larger $H_{\mathrm{rev}}$, and $H_{\mathrm{rev}}$ reduction initially induces a gradual linear shift. However, with reducing $H_{\mathrm{rev}}$, $f_{\mathrm{res}}\left(H\right)$ becomes progressively nonlinear – exhibiting a sharp upturn, followed by a downturn, and finally by another upturn – giving a “W” shaped resonance for lower $H_{\mathrm{rev}}$.

**FIGURE 6:**
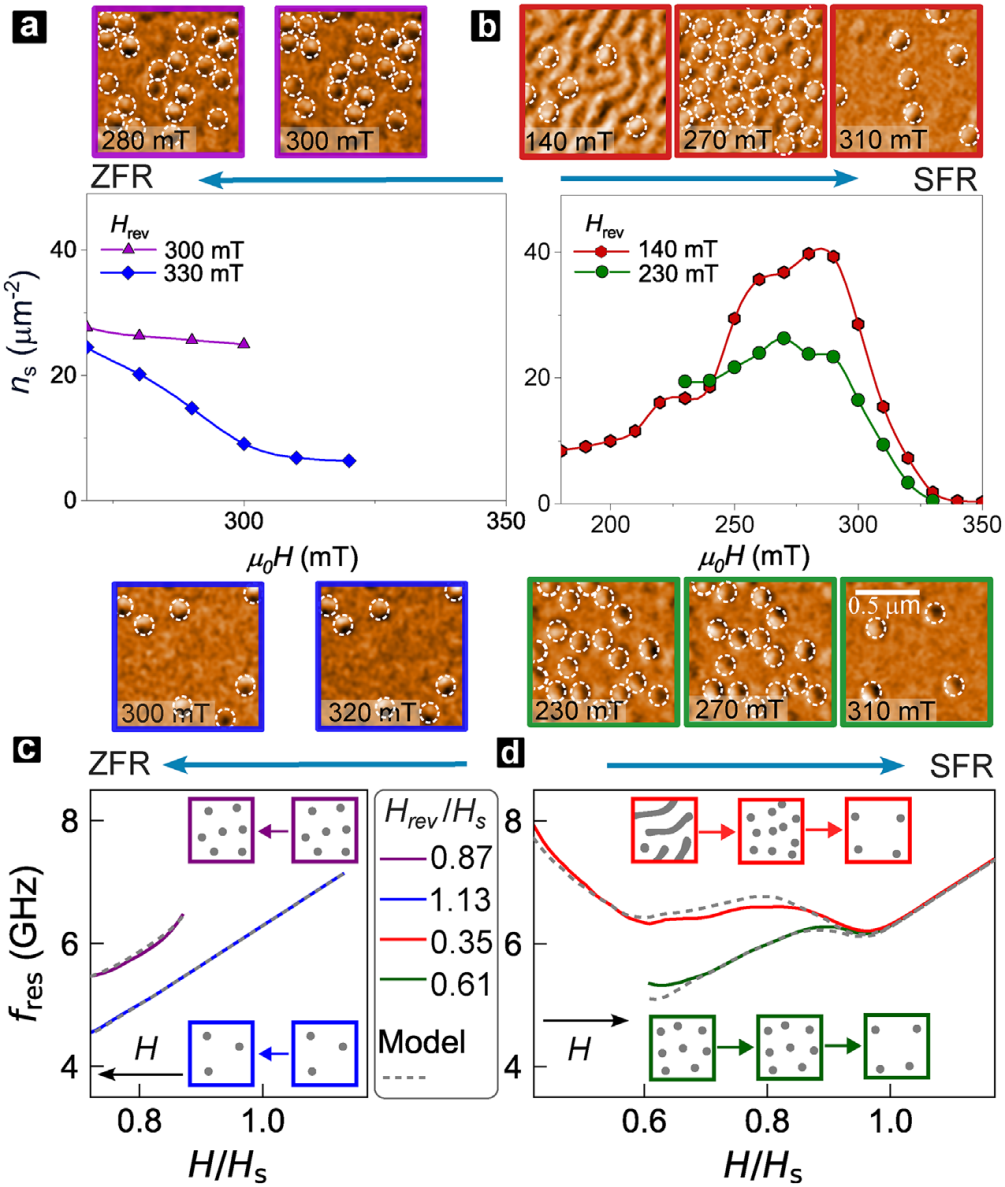
Field Reversal Texture Evolution & Resonances. (a,b) LTEM‐measured skyrmion density, $n_{S}\left(H\right)$, for two representative $H_{\mathrm{rev}}$ values for (a) ZFR and (b) SFR protocols (Figure 5a,b). Insets (top, bottom): representative LTEM images for each $H_{\mathrm{rev}}$ sweep. Arrows indicate field sweep direction, dashed circles identify skyrmions. (c,d) Resonance spectra, $f_{\mathrm{res}}\left(H\right)$ for the two $H_{\mathrm{rev}}$ regimes from simulations (solid lines) and toy model (dashed lines) for (c) ZFR and (d) SFR. Insets show schematics of texture evolution for (c) ZFR (skyrmions) and (d) SFR (stripe fission and/or skyrmion annihilation).

To elucidate the origin of exceptional tunability of $f_{\mathrm{res}}\left(H\right)$ spectra, we performed LTEM imaging (Figure 6a,b) and micromagnetic simulations (Figure 6c,d) for varying $H_{\mathrm{rev}}$ following ZFR and SFR protocols. The ZFR protocol primarily involves isolated skyrmions (density, $n_{S}$) over the relevant field range (Figure 6a). Here, reducing $H_{\mathrm{rev}}$ increases the initialized $n_{S}$, with minimal changes in $n_{S}\left(H\right)$ across the range of H‐sweep (Figure 6a). Thus, while variation of initialized $n_{S}$ renormalizes the Kittel mode (slope and offset, Figure 6c), the qualitative shape of $f_{\mathrm{res}}\left(H\right)$ remains unchanged. In contrast, for SFR, the initialized state is qualitatively distinct – isolated skyrmions ($H_{\mathrm{rev}}∼H_{S}$, Figure 6b: bottom) or a mixed stripe‐skyrmion ensemble ($H_{\mathrm{rev}}\ll H_{S}$, Figure 6b: top), each of which exhibits distinct field evolutions (Figure 6b).

For large $H_{\mathrm{rev}}$, SFR spectra correspond to progressive annihilation of isolated skyrmions, resulting in quasi‐linear dispersion (Figure 6d: green). For low $H_{\mathrm{rev}}$, SFR spectra also involve stripe‐to‐skyrmion fission, and the persistence of a dense skyrmion lattice, prior to their annihilation into the uniform state. This non‐monotonic texture evolution corresponds to down‐up‐down‐up resonance features, forming the “W”‐shaped resonance spectrum (Figure 6d: red). Crucially, our analytical model (Equation (1)) provides the quantitative framework enabling predictive control of these reconfigurable resonance spectra. When applied to simulated CSTs, the model exhibits excellent correspondence with $H_{\mathrm{rev}}$ trends of $f_{\mathrm{res}}\left(H\right)$ for both ZFR and SFR protocols (Figure 6c,d). This cements one‐to‐one correspondence between CST configurations and resonance spectra, and their facile tunability with $H_{\mathrm{rev}}$, which acts as a bespoke, in situ linear or non‐linear knob.

## 6 Outlook

Our work unveils the robust broadband microwave resonance spectrum of minimally damped multilayer CST ensembles at RT. Each irreversible spin‐textural transition generates a Y‐shaped dispersion feature, with distinctive attributes compared to prior material systems. First, the unique thermodynamics of multilayer CSTs yields multiple such features, with field asymmetries due to the interplay of anisotropy and chirality. Second, unlike the narrow‐band nature of localized resonances, the inter‐textural resonances prominent in our work persist over a much broader range of fields and frequencies. Third, their direct relationship to the underlying CST configuration can be remarkably well‐described by a simple quantitative model. Crucially, the ease‐of‐control of multilayer CST configurations enables long‐sought in situ tunability of their dispersion, with desired linear, or non‐linear response to the control knob over the same field and spectral range [35, 36].

The presented ease of microwave programmability of CST ensembles is particularly attractive given their resemblance to MCs [12, 20, 21, 42, 43, 44]. While magnon dispersions are typically configured by nanopatterning in MCs and waveguides [4, 5, 6, 7, 45], both the magnitude and character of CST dispersion exhibit wide‐ranging analog control within as‐deposited films. While we employ reversal field as the modulating knob in this work, CST configurations can be all‐electrically tailored using current or thermal inputs [36, 46, 47]. Such a CMOS‐compatible platform with reconfigurable resonant microwave characteristics may find a wide range of applications in RF microelectronics, from filters to transmitters and multiplexers [1, 11, 12, 20, 22, 44]. Specifically in RF filter design, our work directly enables efficient, scalable, and tunable RF filters suitable for on‐chip microelectronics compared to state of the art technology [48, 49, 50, 51, 52]. Finally, such GHz resonances are finding increasing relevance in unconventional computing applications [8, 26, 45, 53, 54]. The robust memory characteristics of CST ensembles and their (non‐)linear response to an input knob, is particularly attractive for reservoir and stochastic computing architectures [25, 54].

## 7 Experimental Section

### 7.1 Film Deposition

Multilayer films of: Ta(4)/Pt(5)/[Ir(1)/Fe(0.3)/Co(0.7)/Pt(1)]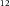/Pt(2) (nominal layer thickness in nm in parentheses, active stack in bold) were deposited on thermally oxidized 200 nm Si wafers by ultrahigh vacuum magnetron sputtering using the Singulus Timaris physical vapor deposition system. Additional multilayer films were prepared for validation studies, whose compositions are detailed in Section S1. For direct comparisons between MAS and LTEM measurements, the films were simultaneously deposited on 20 nm‐thick SiO_2_ membrane grids from SPI Supplies. The structural and magnetic properties of the multilayer stacks are in line with our previous works [33, 55], and the measured magnetic parameters are detailed in Section S1.

### 7.2 Magnetic Parameters

Magnetization measurements, M(H), were performed using a MicroMag Model 2900 alternating gradient magnetometer across OP and IP orientations to determine $H_{S}$, $M_{S}$, and $K_{\mathrm{eff}}$ (details in Section S1).

BLS spectroscopy experiments were performed to quantify the iDMI using a wave‐vector resolved set‐up, upon saturating the samples with an IP magnetic field. BLS spectra were acquired by illuminating the samples with a 532 nm laser beam, and collecting thermally excited Damon‐Eshbach spin waves in back‐scattering geometry with a six‐pass tandem Fabry‐Perot interferometer. iDMI‐induced spin wave non‐reciprocity manifests in BLS spectra as a frequency shift for created (Stokes) and annihilated (anti‐Stokes) magnon peaks. The asymmetric frequency shift, $f{\left(k\right)}_{\mathrm{AS}}$, was quantified by Lorentzian fits to the BLS spectral peaks at the applied field, and upon reversing the magnetic field (Figure 1a) The $f{\left(k\right)}_{\mathrm{AS}}$ thus obtained is related to iDMI as [32]

(2) $$f{\left(k\right)}_{\mathrm{AS}}=\frac{1}{2}\left(f(-k)-f(k)\right)=\frac{\gamma D}{\pi M_{S}}k\;$$

where k and f(k) are the wave vector and frequency of the magnon, and γ is gyromagnetic ratio (obtained from FMR, see Section S1).

### 7.3 Lorentz Microscopy

LTEM experiments were performed using an FEI Titan S/TEM operated in Fresnel mode at 300 kV. A Lorentz lens was used with a defocus of −2.4 mm, and samples were tilted at $15^{\circ }$ relative to normal incidence to ensure sufficient contrast for Néel domains [31, 36]. To study the field evolution of CSTs, an OP field was applied by energizing the objective lens at sample position. LTEM images were subsequently acquired using field intervals and protocols similar to those for MAS. Due to OP field strength limitations for negative values below ‐0.3 T, imaging for such cases were performed by flipping the polarity of the sample or the field protocol, as relevant. LTEM images were processed using a background subtraction protocol, detailed in our previous works [31, 36]. The densities of skyrmion and stripe textures were evaluated by manual identification and counting from LTEM images recorded at respective fields (see Section S2).

### 7.4 MAS Experiments and Analysis

Broadband MAS measurements were performed using two home‐built setups used for high‐resolution spectroscopy of ultrathin magnetic films [56]. A 4×8 mm blanket sample coupon was inductively coupled to a U‐shaped CPW using a spring‐loaded sample holder. Microwave excitations were sourced parallel to the sample plane (Figure 2a, inset: $h_{\mathrm{rf}}$), and the transmitted signal ($S_{21}$) was measured using Keysight N5222 & N5232A vector network analysers (VNA). External OP magnetic fields up to ±1 T were applied to saturate the sample, and data were recorded at each field in frequency sweep mode (e.g., Figure 2b), and separately also at each frequency in field sweep mode (e.g., Figure 2c), over frequencies of 1–26 GHz. The acquired MAS spectra were analysed after subtracting the CPW background recorded without any samples placed using the same measurement protocol (details in Section S3). For the two field‐reversal protocols (SFR and ZFR), MAS spectra were recorded in frequency sweep mode with OP field swept from reversal field to saturation and ZF, respectively. Representative MAS spectra for these protocols are shown in Section S4, Figure 5c,d show plots of the resonant peak positions $f_{\mathrm{res}}\left(H\right)$ for each $H_{\mathrm{rev}}$. The resonant peak positions are identified by fitting the MAS spectra to a Lorentzian function $\left(\frac{A}{{\left(f-f_{\mathrm{res}}\right)}^{2}+w^{2}}\right)$ (Figure 5).

### 7.5 Micromagnetic Simulations

Micromagnetic simulations of CST evolution and microwave response were performed using the finite‐element mumax^3^ package [57] using a mesh size of 4×4×3 nm with 512×512×1 cells. The Ir/Fe/Co/Pt stack was modelled as an effective medium layer [15], while the repetition of the stack was considered by introducing periodic boundary condition in z direction (4 repeats each along ±z). The magnetic parameters used are detailed in (Section S5) and are overall consistent with experiments. Random grains were introduced to emulate nanoscale magnetic variations, wherein iDMI and $K_{\mathrm{eff}}$ were varied across grains following standard micromagnetic protocols [58]. The hysteresis loop was simulated following recipes used across our previous works [31, 35, 36]. MAS were simulated by examining the response of IP magnetization to a spatially uniform AC excitation field

(3) $$h\left(t\right)=h_{0}\sin\left(2\pi f_{c}t\right)/\left(2\pi f_{c}t\right)\;$$

where $h_{0}=10$ mT, and $f_{c}=50$ GHz determines the simulation bandwidth. The temporal response of average magnetization were recorded for 10 ns, and Fourier transformed to obtain the MAS spectrum. This procedure was separately applied to each magnetization configuration in Figure 3a,b to obtain Figure 3c. Spatially resolved microwave absorption were studied by introducing a sine wave IP excitation with amplitude 0.2 mT. The sinusoidal excitation frequency used was determined from the MAS peak positions. The absorption amplitude was evaluated by time‐averaging over the temporal response as:

(4) $$\delta m^{2}\left(x,y\right)={\langle {\left[m\left(x,y,t\right)-{\langle m_{0}\left(x,y\right)\rangle }_{t}\right]}^{2}\rangle }_{t}$$

where t denotes the time average over 10 ns.

### 7.6 Toy Model

To quantify the resonance phenomenology of CSTs, we have proposed a toy model. The high‐frequency modes (CF, KM) are modelled as 2D standing magnon modes, wherein nodal lines are defined by CST domain boundaries, as shown in Figure 4g: inset. The resonance frequency is then given by

(5) $$f\left(H\right)=\frac{C}{d_{\mathrm{avg}}^{2}}+f_{K}\left(H\right)\;$$

Here, $d_{\mathrm{avg}}$, the average distance between CSTs, was calculated by averaging the medial axis distance of the uniform background magnetization. Meanwhile, $f_{K}\left(H\right)$ is the linearly dispersing uniform magnetization precession for OP configuration, given by the well‐known Kittel equation [37, 38]

(6) $$f_{K}\left(H\right)=\frac{\mu_{0}\gamma }{2\pi }\left(H-M_{\text{eff}\;}\right)\;$$

where γ is the gyromagnetic ratio, and $M_{\mathrm{eff}}$, the effective magnetization, is obtained by fitting the uniform mode dispersion (details in Section S1). Finally, C is a fitted parameter related to the exchange strength, determined by fitting the resonances from the skyrmion lattice ($f_{\mathrm{res}}\left(d_{\mathrm{avg}}\right)$), as shown in Figure 4g. Using these parameters, the resonance peaks were analytically calculated for the magnetization configuration at each field to reconstruct the full microwave spectrum (Figure 4h), thereby establishing the validity of the toy model.

## Conflicts of Interest

The authors declare no conflicts of interest.

## Supporting information


**Supporting File**: adma72421‐sup‐0001‐SuppMat.pdf.

## Data Availability

The data that support the findings of this study are available from the corresponding author upon reasonable request.
