# Supplementary material for: Analog Control of Reconfigurable GHz Resonances from Chiral Spin Texture Ensembles
Source: Adv Mater. 2026 Mar 4;38(18):e21980. doi: 10.1002/adma.202521980 (PMC13014033; doi:10.1002/adma.202521980)
Supplement: Supplementary file 1 — Supporting File: adma72421‐sup‐0001‐SuppMat.pdf. [file ADMA-38-e21980-s001.pdf]

Supporting Information for

Analog Control of Reconfigurable GHz Resonances from Chiral Spin Texture Ensembles

T.S. Suraj,[1] Jifei Huang,[1] Hui Ru Tan,[2] Abhijit Ghosh,[2] Jing Zhou,[2] Hang Khume Tan,[2] May Inn Sim,[1] Alexander K. J. Toh,[2] Xiaoye Chen,[2] and Anjan Soumyanarayanan[1,2,*]

[1]*Department of Physics, National University of Singapore, 117551 Singapore*
[2]*Institute of Materials Research and Engineering, Agency for Science, Technology and Research, 138634 Singapore*

Contents

S1. Magnetic Properties 2

S2. Lorentz TEM Analysis and Complementary Experiments 4

S3. Microwave Spectroscopy Analysis 7

S4. Complementary MAS Experiments 9

S5. Micromagnetic Simulations 12

References 16

S1. Magnetic Properties

Sample	M_S (MA/m)	H_S (OP) (mT)	H_S (IP) (mT)	K_{eff} (MJ/m^3)	iDMI (mJ/m^2)	κ	α	γ (GHz/T)	ΔH_0 (mT)
[Fe(0.3)/Co(0.7)]$_{\times 12}$	1.13	325	150	0.10	-2.60 ± 0.05	1.86 ± 0.16	0.022 ± 0.001	190	38.99 ± 0.78
[Fe(0.2)/Co(0.8)]$_{\times 12}$	1.20	190	500	0.27	-2.20 ± 0.08	0.96 ± 0.09	-	168	-
[Fe(0.3)/Co(0.7)]$_{\times 16}$	1.23	374	289	0.12	-2.53 ± 0.06	1.66 ± 0.14	0.031 ± 0.001	155	109.15 ± 0.11
[Fe(0.2)/Co(0.8)]$_{\times 16}$	1.28	200	520	0.27	-2.20 ± 0.08	0.96 ± 0.09	0.033 ± 0.001	198	69.18 ± 3.71

Table S1. **Magnetic Parameters of Samples.** [Ir(1)/Fe(x)/Co(y)/Pt(1)]$_N$ multilayer samples, identified by their acronym [Fe(x)/Co(y)]$_N$ (layer thicknesses in nm are given in parentheses), and their corresponding magnetic properties: saturation magnetization (M_S), out-of-plane (OP) and in-plane (IP) saturation fields (H_S), effective anisotropy (K_{eff}), interfacial Dzyaloshinskii–Moriya interaction (iDMI), chiral domain stability parameter (κ), effective damping (α), gyromagnetic ratio (γ) and inhomogeneous linewidth broadening (ΔH_0)

.

Magnetic Characterization. The static and dynamic magnetic parameters for the four multilayer stacks studied in this work – [Ir/Fe(0.3)/Co(0.7)/Pt]$_{\times 12}$, [Ir/Fe(0.2)/Co(0.8)/Pt]$_{\times 12}$, [Ir/Fe(0.3)/Co(0.7)/Pt]$_{\times 16}$, and [Ir/Fe(0.2)/Co(0.8)/Pt]$_{\times 16}$ - were systematically measured using different experimental techniques, and are listed in Table Tbl. S1. These stack configurations are subsequently represented as Fe(x)/Co(y). Static magnetic parameters such as saturation magnetization (M_{s}), effective anisotropy (K_{eff}), and saturation field (H_{k}) were extracted using AGM magnetometry measurements (see Fig. S1(a,b)). The effective anisotropy (K_{eff}) of the multilayers was determined using the in-plane (IP) anisotropy field, H_{k}, following established methods[1]. The dynamic properties – Gilbert damping (α) and gyromagnetic ratio (γ), were determined from microwave absorption spectroscopy (MAS, see below). Finally, the chiral domain stability parameter (κ) which governs the energetics of spin textures, is defined as

$$\kappa = \frac{\pi D}{4\sqrt{A K_{\text{eff}}}} \tag{S1}$$

The exchange stiffness (A) used in evaluating κ is consistent with our previous report[2].

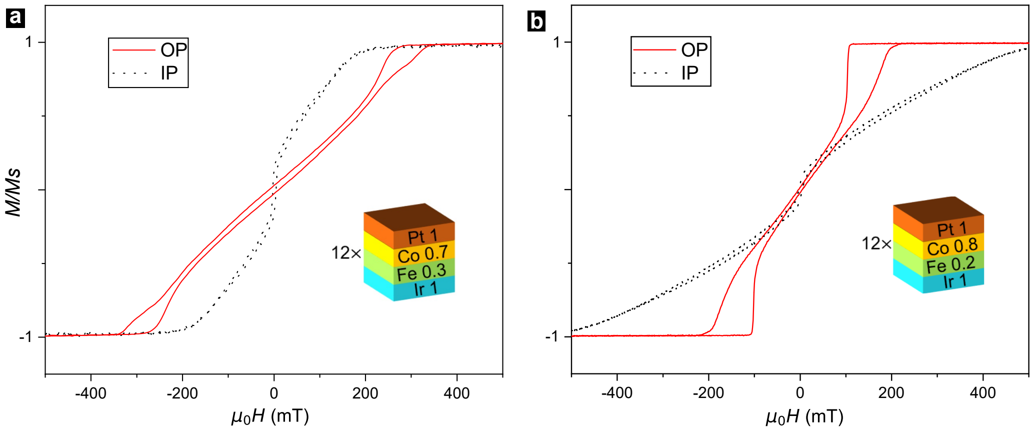

Figure S1. **Measured Hysteresis Loops.** Normalized OP (red) and IP (black) magnetization hysteresis loops, $M(H)/M_{\text{s}}$ for the two samples **(a)** Fe(0.3)/Co(0.7) and **(b)** Fe(0.2)/Co(0.8). Insets show stack configurations.

Dynamic Magnetic Parameters. We evaluated the dynamic magnetic parameters by measuring the microwave transmission parameter S_{21} above saturation, with an out-of-plane (OP) magnetic field swept over $330 - 500$ mT – over a frequency (f) range of $1 - 26$ GHz. Representative spectra are shown in Fig. S2(a–c). To accurately locate the peak position and linewidth, we fit the imaginary and real parts of S_{21} with a standard Lorentzian function following established protocols[3,4] [Fig. S2(d)].

To extract gyromagnetic ratio γ, we fit the dispersion of peak position (H_{res}–f), shown in Fig. S2(e), with the Kittel formula for OP geometry[5]:

$$f = \frac{\mu_0 |\gamma|}{2\pi} \left(H_{\text{res}} - M_{\text{eff}}\right) \tag{S2}$$

Here, the effective magnetization $M_{\text{eff}} = M_{\text{S}} - H_{\text{U}}$, where H_{U} is the uniaxial anisotropy field.

Meanwhile, the effective damping (α) was determined by linearly fitting the linewidth ΔH (Fig. S2(f)) using:

$$\Delta H = \frac{4\pi\alpha}{\mu_0|\gamma|} f + \Delta H_0 \tag{S3}$$

Here, ΔH_0 is the inhomogeneous line broadening. The corresponding values obtained from these fits for the samples studied are listed in Tbl. S1.

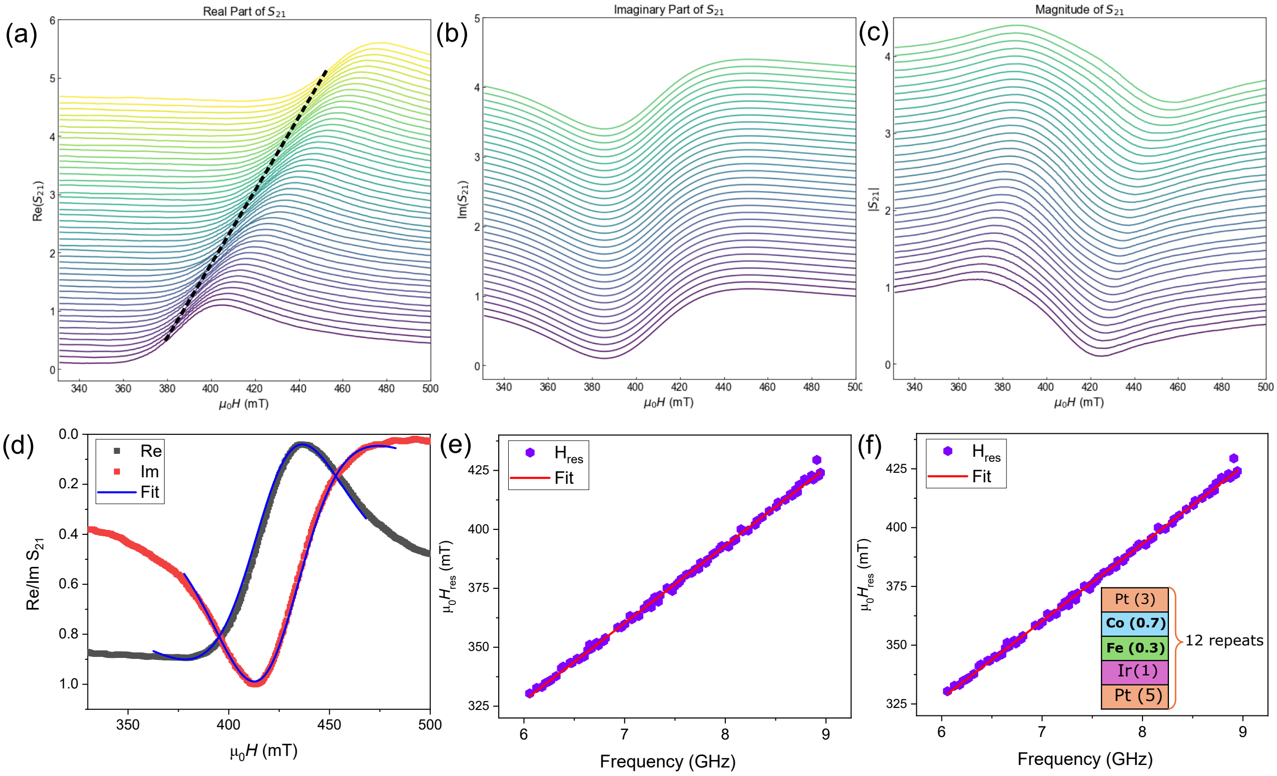

Figure S2. **Uniform (Kittel) resonance mode analysis**. (a-c) Waterfall plots of the (a) real (Re) part, (b) imaginary (Im) part, and (c) magnitude of the complex transmission parameter S_{21}, measured under out-of-plane (OP) magnetic field $\mu_0 H$ (330-500 mT) for sample Fe(0.3)/Co(0.7) (stack schematic: inset of (f)). Data are shown for the field range well above the saturation field, with the evolution of the uniform Kittel mode indicated by the dashed lines in panel (a). (d) Representative complex spectra (Re, Im) at a frequency of 9 GHz fitted with a Lorentzian function (blue). (e–f) Resonance field, $\mu_0 H_{\text{res}}$ (e), and linewidth ΔH (f) plotted as a function of frequency. Linear fits (red) using the Kittel formula (Eq. S3) are used to extract the inhomogeneous broadening ΔH_0 and damping (α), respectively.

S2. Lorentz TEM Analysis and Complementary Experiments

Tilt-Dependent LTEM Study of Texture Chirality. To directly confirm the chiral nature of spin textures in Ir/Fe/Co/Pt multilayers, we performed tilt-dependent Lorentz transmission electron microscopy (LTEM) imaging following established protocols[6,7]. As demonstrated in our foundational works on similar Ir/Fe/Co/Pt multilayers[7], the homochiral Néel textures expected in these samples should not exhibit pronounced contrast at normal beam incidence (zero sample tilt). Meanwhile, finite sample tilt produces antisymmetric contrast about the domain centre arising from the in-plane magnetization component parallel to the electron beam[6]. Fig. S3 shows representative tilt (α)-dependent LTEM images for $[Ir/Fe(0.3)/Co(0.7)/Pt]_{12}$. At zero tilt ($\alpha = 0°$), magnetic contrast is nearly absent, except for slight undulations known to arise from impurities and membrane roughness[7]. Meanwhile, for $\alpha = 15°$, spin textures exhibit pronounced antisymmetric contrast. The LTEM observations are fully consistent with the left-handed Néel chirality inferred independently from Brillouin light scattering (BLS) measurements of interfacial Dzyaloshinskii-Moriya interaction (iDMI) for these films.

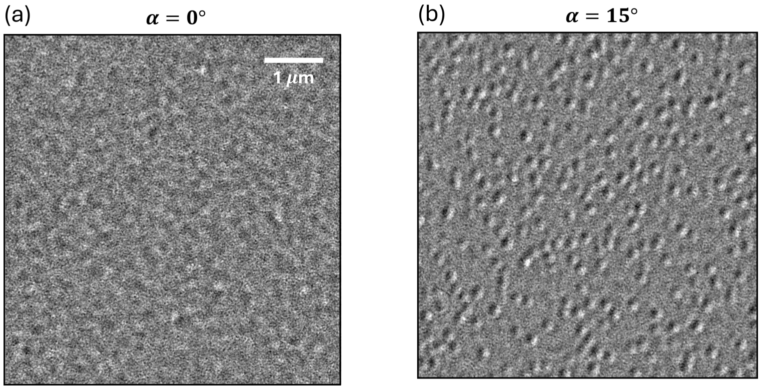

Figure S3. **Tilt-dependent LTEM imaging of texture chirality.** Representative LTEM images of $[Ir/Fe(0.3)/Co(0.7)/Pt]_{12}$ acquired at sample tilt (a) $\alpha = 0°$ (normal incidence) and (b) $\alpha = 15°$. Pronounced antisymmetric spin-texture contrast appears only for finite tilt.

Domain Counting. Manuscript Fig. 1d shows the OP field evolution of skyrmion and stripe densities. To identify and count textures, we first performed a background subtraction on LTEM images to remove contrast originating from non-magnetic sources, using the image at the saturation field as the background[8]. The LTEM-imaged textures were visually distinguished as skyrmions or stripes, and counted over a 2×2 μm region across fields, following recipes detailed in our previous works[7,8]. Fig. S4 shows a representative result of texture identification and counting.

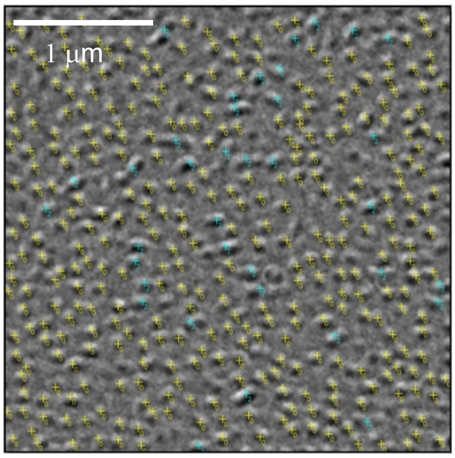

Figure S4. **Texture Identification in LTEM Images.** Representative LTEM image of Fe(0.3)/Co(0.7) at -300 mT (tilt angle: 20°, defocus: -2 mm) used to demonstrate identification and counting of skyrmions (yellow crosses) and stripes (cyan crosses).

Texture Evolution for Varying κ. Manuscript Fig. 1d shows that for Fe(0.3)/Co(0.7), the stripe-skyrmion transitions are distinct for field-polarized and unpolarized sides. Here, we compare the texture evolution of

Fe(0.3)/Co(0.7) with Fe(0.2)/Co(0.8), which have different magnetic parameters and domain stability (Tbl. S1).

For Fe(0.3)/Co(0.7) (Fig. S5(a)), as the field is decreased from the polarized state (+350 mT), skyrmions emerge at 300 mT, with density n_S peaking at $\approx 20\ \mu m^{-2}$ at 250 mT. As the field is swept down, n_S reduces as skyrmions elongate into stripes, which form a labyrinthine configuration at zero field (ZF). For the unpolarized state, as the field swept from ZF to -350 mT, disconnected stripes each fission into multiple skyrmions, resulting in a plateau of high skyrmion density ($n_S \approx 50\ \mu m^{-2}$) at -270 mT. Thus, for Fe(0.3)/Co(0.7), the asymmetric texture evolution and high skyrmion density arise from distinct, irreversible textural transitions.

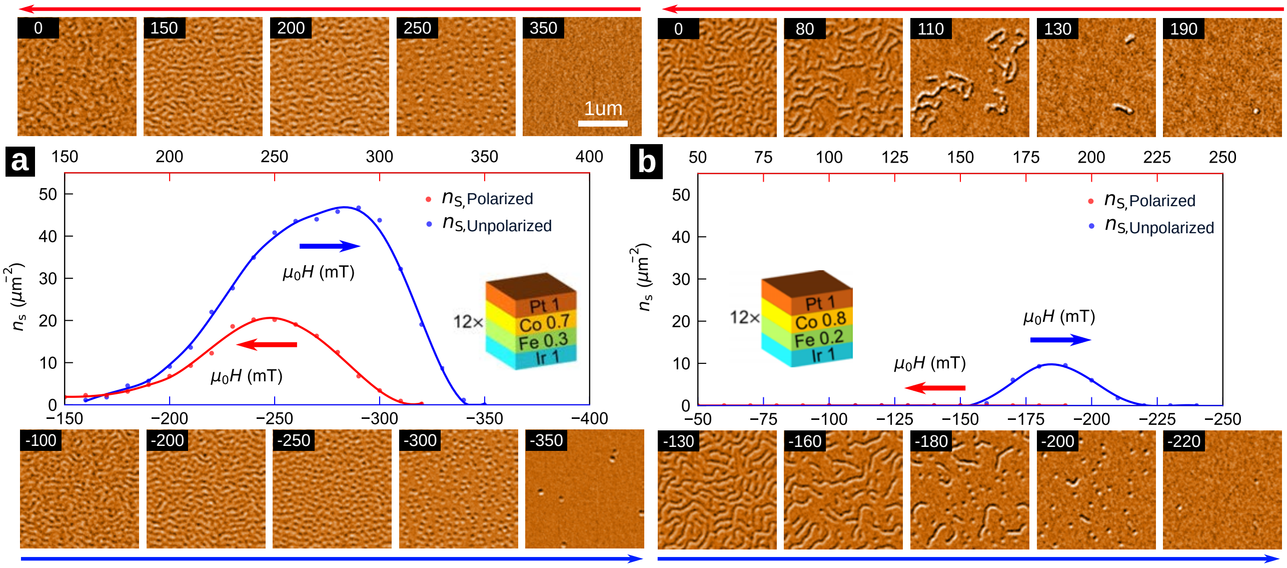

Figure S5. **Texture Evolution for Varying κ.** LTEM images showing the field evolution of skyrmion density, n_S, on field-polarized (red) and unpolarized (blue) sides for (a) Fe(0.3)/Co(0.7) (left) and (b) Fe(0.2)/Co(0.8) (right). Solid lines are guides to the eye. Top (field-polarized) and bottom (unpolarized) panels display representative LTEM images (scalebar: 1 μm) for the sweeps at indicated fields. Red (polarized) and blue (unpolarized) arrows indicate the field sweep direction. Insets show respective stack compositions.

In contrast, for Fe(0.2)/Co(0.8), we observed distinct field evolution of textures (Fig. S5(b)). As the field was decreased from the polarized state (250 mT), no skyrmions were observed. Instead, short stripes emerged and elongated into the labyrinthine state. On the unpolarized side, as the field was swept from ZF to -250 mT, the stripes shrank and transitioned into sparse skyrmions, with a low peak in skyrmion density ($n_S \approx 10\ \mu m^{-2}$) at -180 mT.

The texture transition on the unpolarized side is different for the two samples. For Fe(0.2)/Co(0.8), each stripe shrinks into one skyrmion, whereas for Fe(0.3)/Co(0.7), each stripe fissions into multiple skyrmions. The difference in texture formation mechanism aligns well with the measured skyrmion stability parameter, κ[7–9]. For Fe(0.2)/Co(0.8), $\kappa \simeq 0.9 < 1$, and so a reversible formation stripe-skyrmion transition is anticipated, in line with the observed shrinking of stripes. Conversely, for Fe(0.3)/Co(0.7), $\kappa \simeq 1.8 > 1$, and hence an irreversible stripe-skyrmion transition is expected, consistent with the observed stripe fission.

MFM and LTEM Comparison. To establish the reliability of imaged spin texture configurations (manuscript Fig. 1), key to interpreting MAS resonance modes, we compared LTEM imaging with MFM measurements for an identical $[\mathrm{Ir/Fe(0.3)/Co(0.7)/Pt}]_{\times 8}$ sample at representative OP magnetic fields (Fig. S6). A visual comparison of MFM and LTEM images (Fig. S6(b-c)) indicates comparable spin-texture configurations at similar fields. Notably, quantitative analyses of skyrmion density (Fig. S6(d)) revealed near-identical magnitudes and field-evolution trends for both MFM and LTEM - including across the hysteretic ("YY" region) of interest to this work (Fig. S6(a)). Additionally, at lower fields, the stripe periodicity also agrees remarkably well across MFM (0.120 μm) and LTEM (0.121 μm). In conjunction with our previous works[7,8], this validates the use of LTEM as the primary tool for quantifying the field evolution of spin-texture density in this work.

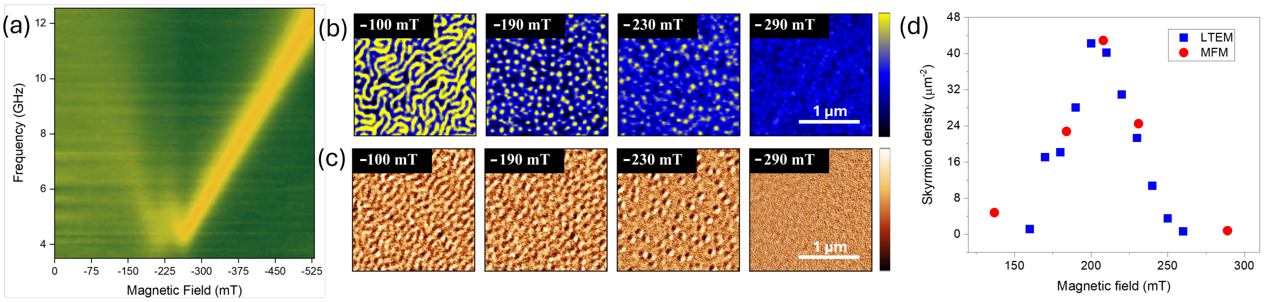

Figure S6. **Comparison of LTEM and MFM of spin textures for** [Ir/Fe(0.3)/Co(0.7)/Pt]$_{\times 8}$**.** (a) MAS colour plot of normalized transmission (S_{21}) against frequency (f) and out-of-plane (OP) magnetic field ($\mu_0 H$), showing hysteretic ("YY") resonances for unpolarized sweep. (b–c) Representative MFM (b) and LTEM (c) images of spin texture evolution for varying unpolarized OP fields. (d) Comparison of field evolution of skyrmion density (n_{S}) extracted from LTEM (blue squares) and MFM (red circles).

S3. Microwave Spectroscopy Analysis

CPW Background Removal Protocol. Within broadband flip-chip spectroscopy, accurate determination of the microwave response of magnetic thin films, including multilayers, is challenging due to large background noise. This is because signal-to-noise ratio (SNR) is limited by the magnetic moment of the film relative to background contributions. The measured transmission parameter S_{21}, includes contributions from the microwave circuit, e.g., cables, connectors, adapters, non-magnetic materials (e.g. substrate), and calibration protocols, which together constitute the non-magnetic background transmission, S_{21}^0. The measured S_{21} is thus related to magnetic susceptibility, $\chi(H)$, of the material of interest via:

$$S_{21}(H) = S_{21}^0 - \frac{\chi(H)}{\tilde{\chi}_0} \tag{S4}$$

We employ a background removal method to obtain the signal of interest, whose magnitude is approximately

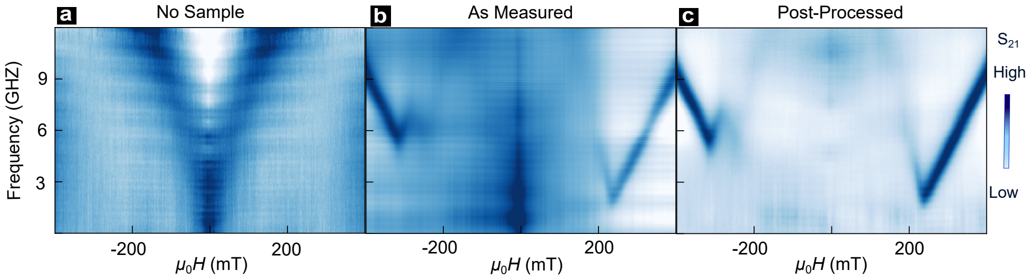

Figure S7. **CPW Background Removal.** Colour plots of normalized S_{21} against frequency and OP field, measured using the frequency sweep protocol, for (a) as-measured data on bare co-planar waveguide (CPW), i.e., without sample loaded; (b) as-measured data for Fe(0.3)/Co(0.7); and (c) post-processed data for Fe(0.3)/Co(0.7) after CPW background removal (see text for details).

1% of the low-frequency background contribution. The background signal $S_{21}^0(f, H)$ (Fig. S7(a)) has a clear frequency dependence characteristic of microwave circuits – larger losses are observed at higher frequencies. Normalizing by field removes this field-independent term. However, the CPW also generates a field-dependent background signal, which is prominent for low SNR samples, such as in our case (Fig. S7(b)). This field-dependent background can be removed by careful subtraction of data acquired without a loaded sample using the identical sweep protocol and measurement parameters (Fig. S7(a) c.f. (b)).

To remove the background component from the data of interest, we follow the steps below:

1. Both sample and background datasets are acquired using identical sweep protocols and measurement parameters.
2. Both datasets are first denoised by a Savitzky–Golay filter.
3. A line-by-line spectral subtraction is performed between the original signal (with sample, Fig. S7(b)) and the background signal (without sample, Fig. S5(a)).
4. This subtracted data is then normalized.

The data resulting from this procedure has minimal CPW background contribution, and highlights the microwave response of the magnetic film (Fig. S7(c)).

Comparison with Derivative-Divide Protocol. For our work, we have performed background noise removal from the MAS data using two distinct measurements (with and without sample). Several background removal methods have been used across literature for MAS data, of which the derivative divide (DD) method is frequently employed[10]. This method involves calculating the numerical derivative of the S_{21} spectra with respect to the magnetic field, followed by normalization to the microwave transmission, which effectively removes the non-magnetic frequency-dependent transmission background. This approach is easier to implement than our "bare CPW" background removal procedure, as it does not require separate microwave circuit calibration. Consequently, it has been used to remove background artifacts in chiral magnetic films with lower SNR[11,12]. To benchmark our background removal technique, we applied the DD method to both field sweep (Fig. S8(a)) and frequency sweep (Fig. S8(b)) MAS S_{21} spectra acquired on Fe(0.3)/Co(0.7) sample (manuscript Fig. 2(b,c)). Fig. S8(a-b) show that the DD plots clearly resolve the key resonant features with distinct Y and YY shapes for polarized and unpolarized sides, with increased SNR relative to our approach (manuscript Fig.2(b,c)). However, the DD method alters the lineshape of the resonances, as the derivative of S_{21} spectra replace the resonance peak with a dip-hump feature.

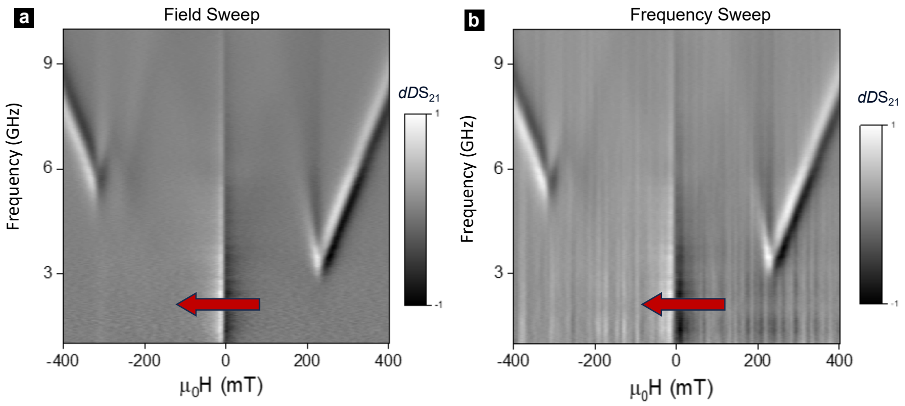

Figure S8. **Derivative Divide (DD) MAS Analysis.** Normalized 2D (f, H) plots of MAS spectra of Fe(0.3)/Co(0.7) subjected to DD analysis (dDS_{21}) for background removal, acquired using (a) field-sweep (constant-f) and (b) frequency sweep (constant-H) protocols, respectively. Red arrows indicate direction of field progression.

Such lineshape changes can potentially distort the finer details of resonance peaks, complicating the analysis and interpretation of resonance modes, their origin and dispersion. In contrast, our background removal technique can also effectively resolve all resonant modes, crucially without altering the resonance lineshape. Therefore, we opted not to use the DD protocol in our manuscript.

S4. Complementary MAS Experiments

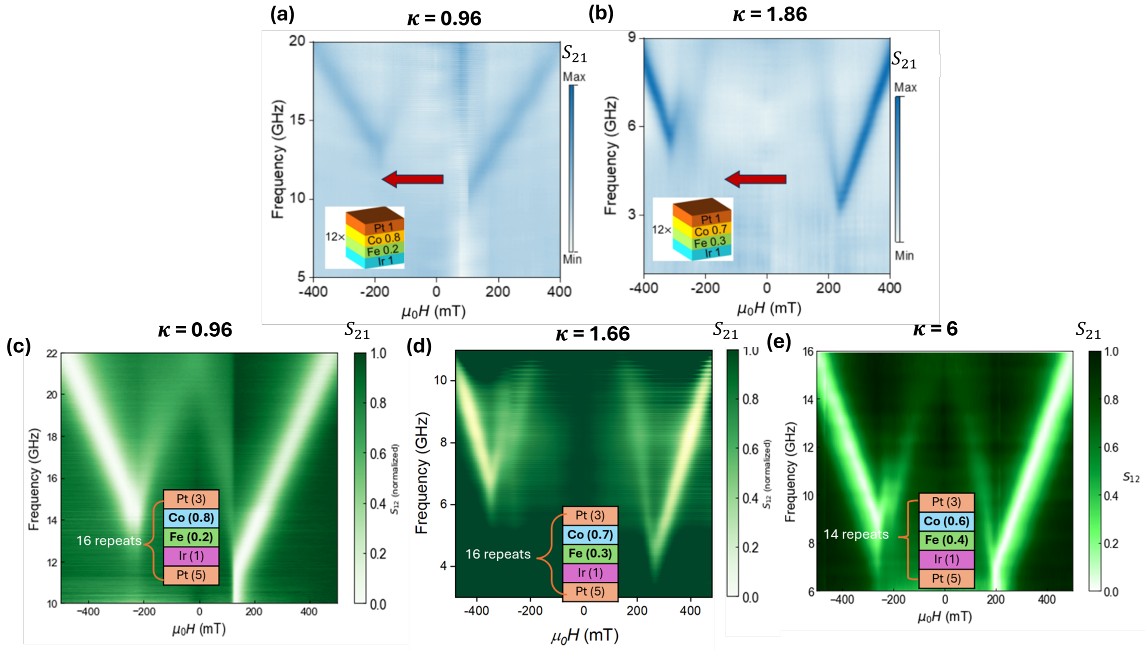

Figure S9. **MAS plots with varying texture stability (κ).** Experimentally measured colour plot of normalized transmission (S_{21}) against frequency (f) and out-of-plane (OP) magnetic field ($\mu_0 H$) for (a) $[\mathrm{Fe}(0.2)/\mathrm{Co}(0.8)]_{12}$ ($\kappa \sim 0.96$); (b) $[\mathrm{Fe}(0.3)/\mathrm{Co}(0.7)]_{12}$ ($\kappa \sim 1.86$); (c) $[\mathrm{Fe}(0.2)/\mathrm{Co}(0.8)]_{16}$ ($\kappa \sim 0.96$); (d) $[\mathrm{Fe}(0.3)/\mathrm{Co}(0.7)]_{16}$ ($\kappa \sim 1.66$); and (e) $[\mathrm{Fe}(0.4)/\mathrm{Co}(0.6)]_{14}$ ($\kappa \sim 6$). The resonances are distinct (similar) for both sides of ZF, i.e., YY vs. Y-shaped (consistently Y-shaped) for all samples with $\kappa > 1$ ($\kappa < 1$).

Evolution of Hysteretic Mode with Material Parameters. To convincingly establish the influence of textural transitions on the MAS resonance modes, it is crucial to compare the resonance characteristics for varying κ. As discussed in SI § S2, Fe(0.2)/Co(0.8) ($\kappa \simeq 0.9$) exhibits a contrasting field evolution of textures compared to the sample of interest – Fe(0.3)/Co(0.7) ($\kappa \simeq 1.8$). Therefore, we performed MAS measurements on Fe(0.2)/Co(0.8) using similar protocols, for direct comparison with Fe(0.3)/Co(0.7) (Fig. S9). For both samples, the finite K_{eff} accounts for the mode renormalization to higher frequencies on the unpolarized side[13].

Meanwhile, the MAS spectra of Fe(0.2)/Co(0.8) (Fig. S9(a)) exhibits several distinctive features when compared with Fe(0.3)/Co(0.7) (Fig. S9(b)). First, the 2D MAS plots for Fe(0.2)/Co(0.8) appear more symmetric about ZF. On the polarized side, as the OP field is swept from $+H_{\mathrm{S}}$ to ZF, the Kittel mode (KM) weakens and abruptly transitions into a confinement (CF) mode, giving a "V"-shaped resonance feature, unlike the "Y" shaped resonance feature of Fe(0.3)/Co(0.7). The absence of the gyrotropic (GY) skyrmion mode is in agreement with the textural evolution of Fe(0.2)/Co(0.8) (Fig. S5), wherein no skyrmions were observed on the polarized side. Meanwhile, on the unpolarized side, a conventional 'Y' shaped resonance feature is observed near saturation, as expected for the annihilation of isolated skyrmions. Notably, the 'YY' shaped feature observed for the unpolarized side of Fe(0.3)/Co(0.7), arising from the irreversible stripe-skyrmion transition, is absent for Fe(0.2)/Co(0.8).

This conclusively establishes that the hysteretic resonance features are governed by the irreversibility of textural transitions. The nature and extent of irreversibility depends in turn on κ, as demonstrated in our previous studies[7–9,14].

To provide further experimental validation, we extended the comparison of MAS features to a broader set of κ values using Ir/Fe/Co/Pt multilayers with varying Fe/Co composition and repeats, presented in Fig. S9 (c–e). These additional measurements explicitly demonstrate the systematic evolution of resonance features with κ. For $\kappa < 1$ samples, the MAS f–H plots consistently display a single 'Y'-shaped feature on both sides of ZF. In contrast, for all $\kappa > 1$ samples, the dispersion has a distinct 'YY'-shape on the unpolarized side. This κ-dependent distinction of the MAS dispersion, consistent with our simulations (Fig. S11), provides robust experimental evidence for hysteretic resonances governed by irreversible textural transitions (governed by κ) discussed in the manuscript.

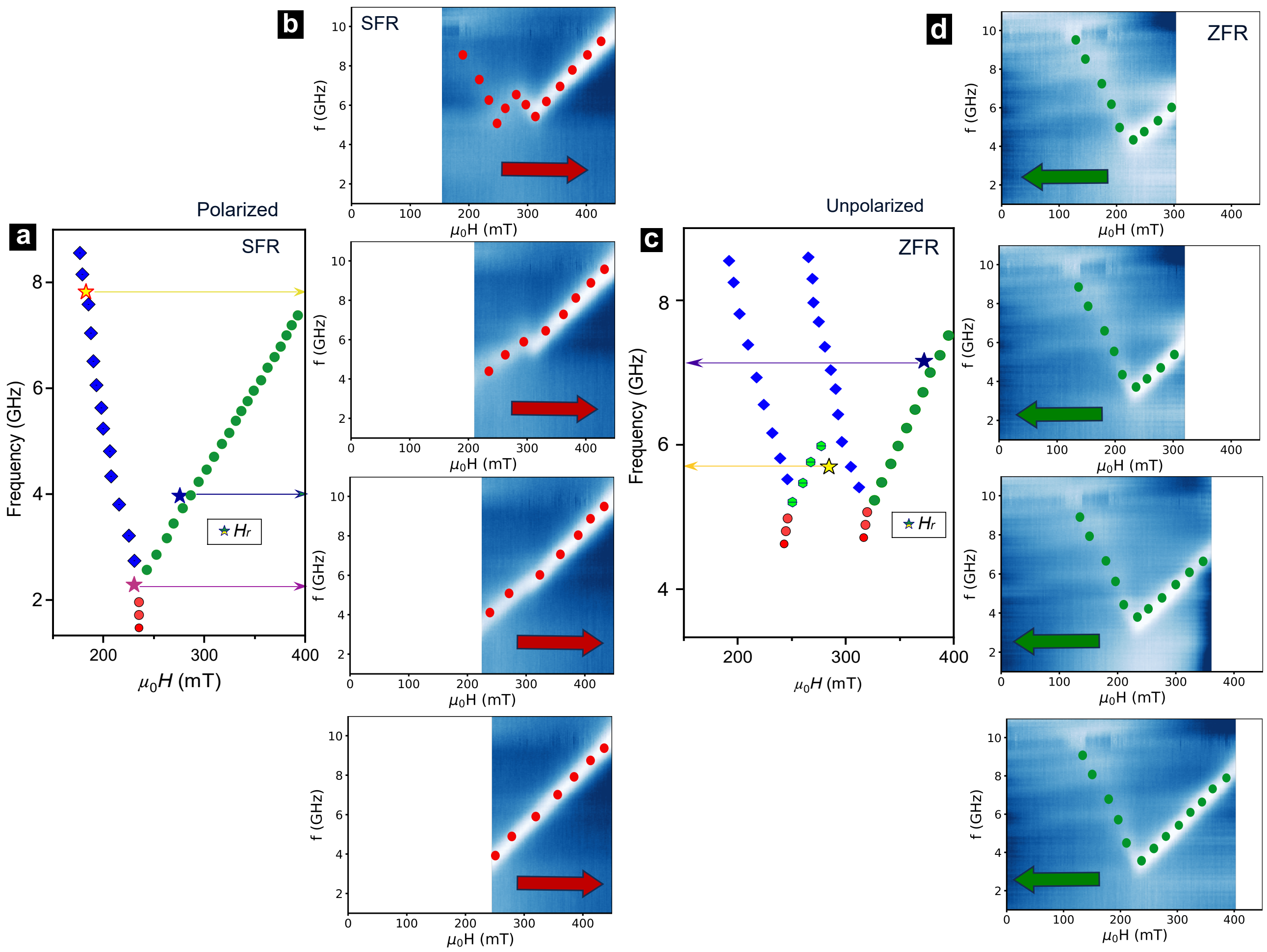

Figure S10. **Field Reversed Resonance Spectra.** (a, c) Skeletal colour-coded MAS resonance plots (from manuscript Fig.1c), used for initializing minor loop spectral sweeps for sample Fe(0.3)/Co(0.7) with reversal field $H_{\rm rev}$ via (a) saturated field-reversal (SFR) and (c) zero-field reversal (ZFR) protocols (see manuscript Expt. Section). (b, d) 2D colour plots of normalized $S_{21}(f, H)$ acquired using frequency sweep protocol for selected $H_{\rm rev}$ values using (b) SFR and (d) ZFR protocols. Solid (red, green) circles indicate resonance peaks, (red, green) arrows represent the field sweep direction, initialized at the respective $H_{\rm rev}$.

Field Reversal MAS Measurements . Following established field reversal magnetometry[7,9,15] and magnetoresistance techniques[16], we have performed MAS measurements with varying minor loop reversal field, $H_{\rm rev}$, swept across polarized (Fig. S10(a)) and unpolarized regimes (Fig. S10(c)). To eliminate field-dependent artifacts, all minor loop sweeps were performed using frequency sweep protocol. Given the distinct texture configurations on polarized and unpolarized sides, we define two field sweep protocols, saturated field reversal (SFR) and zero field reversal (ZFR), both of which involve two steps:

1. **Field Initialization**: To get the desired initial magnetization configuration (polarized or unpolarized).
2. **Sweep Direction**: Recording MAS response as the field is swept from $H_{\rm rev}$ to the final field ($H_{\rm S}$ for SFR, ZF for ZFR).

For ZFR, the field is initialized at $-H_{\rm S}$ and swept through ZF to $H_{\rm rev}$ on the unpolarized side. MAS spectra $S_{21}(f; H, H_{\rm rev})$ are recorded over a sweep from $H_{\rm rev}$ to ZF. This process is repeated by progressively decreasing $H_{\rm rev}$ (1 mT/step) until ZF. For SFR, the field is initialized at $+H_{\rm S}$ and swept to $H_{\rm rev}$ on the same polarized side. MAS spectra $S_{21}^{\rm SFR}(f, H; H_{\rm rev})$ are recorded on the return sweep to $+H_S$. This process is repeated by gradually increasing $H_{\rm rev}$ until $+H_S$. The spectra $S_{21}(f; H, H_r)$ obtained for each $H_{\rm rev}$ are analyzed to locate the resonance peaks $f_{\rm res}$ by fitting to a standard Lorentzian function:

$$|S_{21}(f; H, H_{\rm rev})| = \frac{A}{w^2 + (f - f_{\rm res}(H, H_{\rm rev}))^2} \quad \text{(S5)}$$

The extracted peak positions ($f_{\rm res}$) from corresponding plots are condensed into a single resonance curve, $f_{\rm res}(H)$ (manuscript Fig. 5(c & d)), which describes the fitted peak positions of higher-frequency (CF, KM)

resonance modes. The spectra $S_{21}^{\mathrm{ZFR,\ SFR}}(f, H; H_{\mathrm{rev}})$ for a few representative H_{rev} are shown in Fig. S10(b & d). Here, red (SFR) and green (ZFR) dots show the extracted resonant peaks (f_{res}), along with the skeletal plot of polarized (Fig. S10(a)) and unpolarized (Fig. S10(c)) MAS spectra.

S5. Micromagnetic Simulations

Magnetic Parameters for Simulations. Micromagnetic simulations were performed using mumax[3], as detailed in manuscript (Experimental Section)[17], using the parameters listed in Tbl. S2. The effective medium approximation was applied by treating Fe/Co as a 1 nm thick ferromagnetic (FM) layer, and Ir/Pt as a 2 nm thick non-magnetic layer.

Simulations used an interfacial iDMI of 2.0 $\mathrm{mJ\,m^{-2}}$, which best reproduce the experimentally measured hysteresis loops. For control simulations with $D = 2.6$ $\mathrm{mJ\,m^{-2}}$ (BLS-measured value), the microwave spectra are qualitatively consistent with experiments, albeit with quantitative differences. The simulations incorporate $\pm7\%$ granular variation in magnetic properties[18,19] to account for the polycrystallinity of our samples, as typically used to model dynamic magnetic properties. A higher damping ($\alpha = 0.05$) was used to speed up the relaxation without visibly altering the final state. The saturation field for simulations is $H_\mathrm{S} = 220$ mT. The quantitative differences with experiments can be ascribed to the shorter simulation time (10^{-5} s, vs. $\sim 10^2$ s in experiments), and the ensuing requirement of stronger excitation that enables visualization of key trends.

	M_S (MA/m)	K_u (MJ/m^3)	A (pJ/m)	D (mJ/m^2)	α
Values	1.36	1.16	15	2	0.05
Variation in Grains	-	7%	-	7%	-

Table S2. **Simulation Parameters.** Magnetic parameters and their granular variation used for micromagnetic simulations of Fe(0.3)/Co(0.7) equivalent stacks.

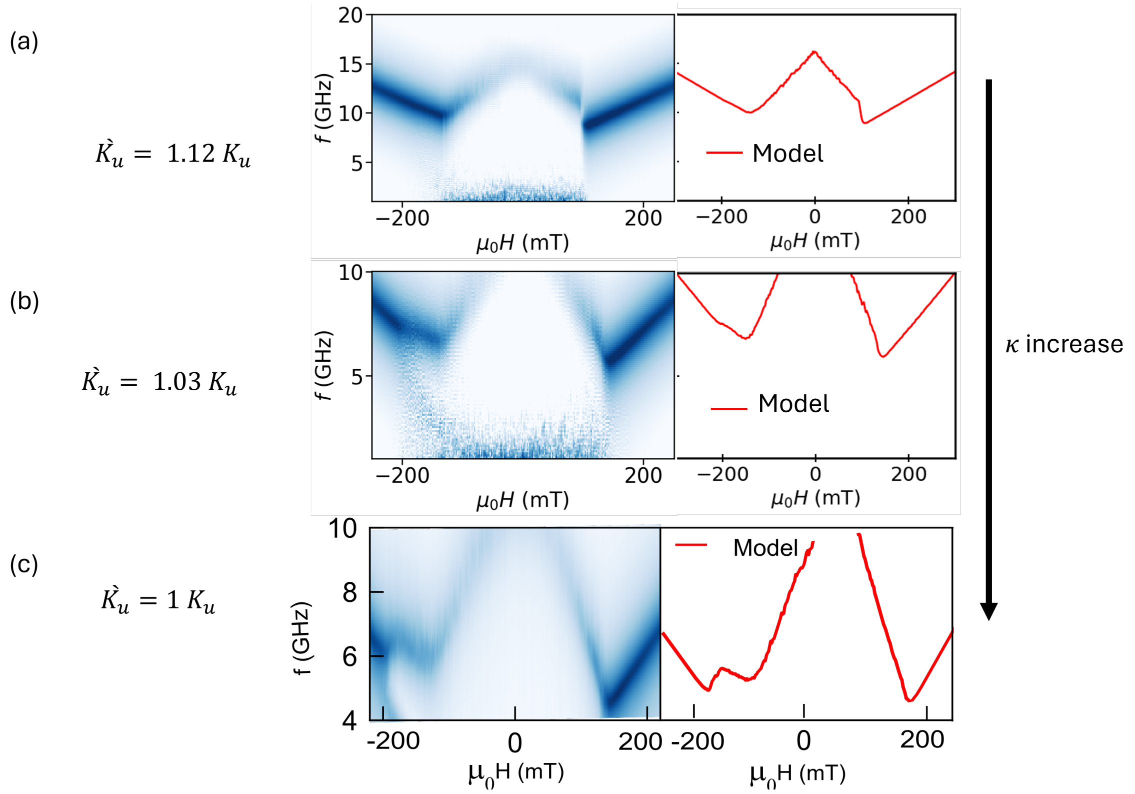

Figure S11. **Simulated Resonances for Varying Anisotropy.** Simulated MAS colour plots (left, c.f. manuscript Fig. 3(c)) and toy model (Eqn. 1, Manuscript) predicted resonance modes (right) from simulated spin-texture configurations (c.f. manuscript Fig. 4(h)) for stack parameters emulating experiments, with decreasing anisotropy (K_u) from top to bottom: (a) $K'_u = 1.12\,K_u$, (b) $K'_u = 1.03\,K_u$, and (c) $K'_u = 1.00\,K_u$.

Simulated κ Evolution of Hysteretic Resonance. To evidence the experimentally observed evolution of the resonance features with κ, we performed additional simulations (Fig. S11, left) in which κ was lowered by incrementally increasing the uniaxial anisotropy $\left(K_u = K_\mathrm{eff} + \mu_0 M_S^2/2\right)$, while keeping all other parameters unchanged. Recall that $\kappa = \frac{\pi D}{4\sqrt{A K_\mathrm{eff}}}$, so κ decreases as K_eff (and thus K_u) increases. As κ is reduced, the "YY" dispersion evolves into a "Y" shape, as observed experimentally. As an aside, the resonances are broader at low K_u, while with increasing K_u they become progressively sharper and more distinct, highlighting the role of anisotropy in defining the resonance features. Finally, the simulated dispersions evolve in close agreement

with the toy-model predictions for the respective spin-texture configurations (Fig. S11, right), confirming that the simplified toy model reliably reproduces both the dispersion and the line-shape evolution of the microwave resonances of spin textures.

Domain Counting for Simulated Images. Simulated textures possess large SNR compared to experimental LTEM images, and therefore, algorithmic counting was performed after classifying domains based on their circularity. Here, domain circularity is defined as:

$$\text{Circularity} = \frac{4\pi A}{P^2} \tag{S6}$$

where A is the area and P the perimeter of the domain. Textures with circularity above 0.65 were classified as skyrmions, and the remainder were stripes.

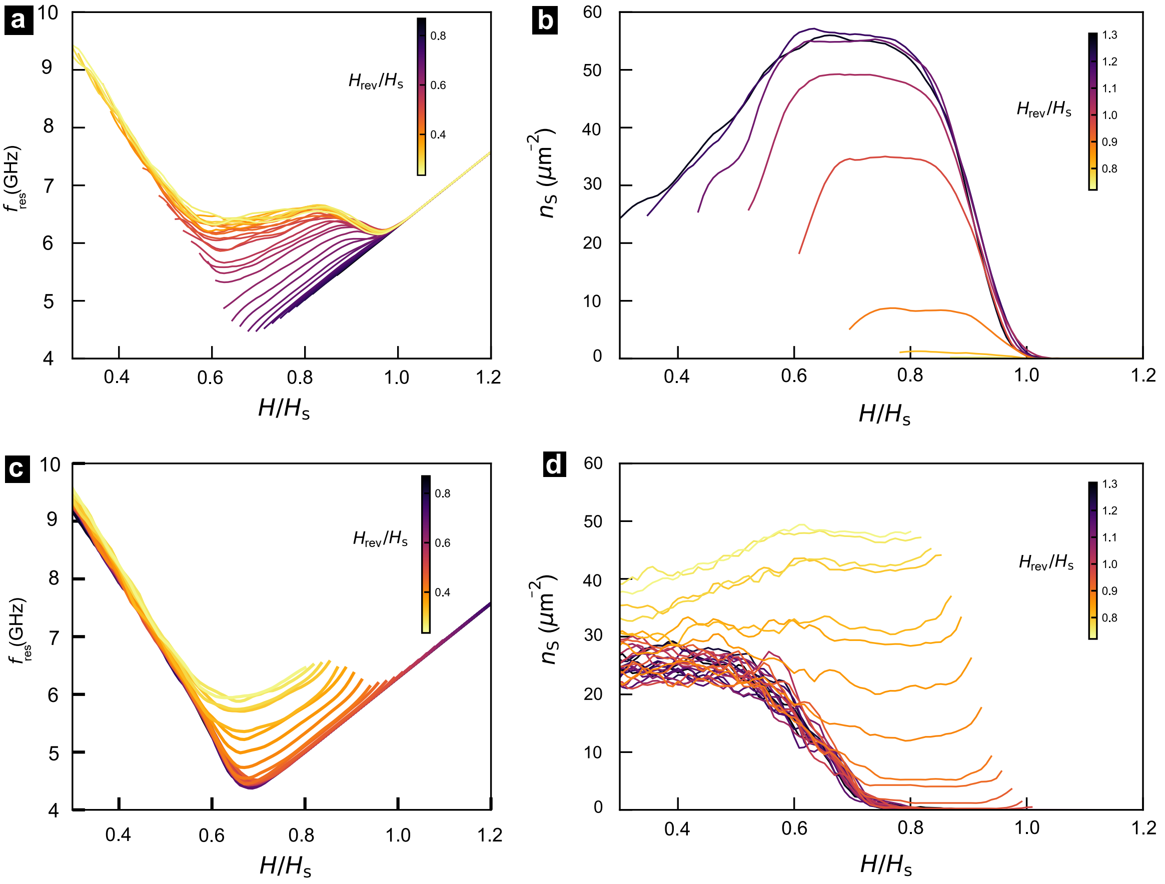

Figure S12. **Simulated Field-Reversed MAS Dispersion and Texture Density.** Micromagnetic simulations of field evolution of (a, c) MAS resonances ($f_{\text{res}}(H/H_{\text{s}})$ and (b, d) skyrmion density, $n_{\text{S}}(H)$ across varying reversal fields, H_{rev}, following (a, b) SFR and (c, d) ZFR protocols (details in manuscript Expt Section).

Field Reversal MAS Simulations. The micromagnetic simulations in manuscript Fig.3 enable conclusive identification of the experimentally observed resonance modes (manuscript Fig. 1(b,c)). To elucidate the experimentally achieved tunability of resonant dispersions (manuscript Fig.5), we simulated the field reversal MAS spectra and texture densities following SFR and ZFR protocols for field-polarized and unpolarized sides respectively. The field-reversed data are generated following the steps below:

1. First, a full hysteresis loop was simulated from $+H_{\text{S}}$ (polarized) to $-H_{\text{S}}$ (unpolarized) (similar to manuscript Fig. 3) to obtain texture configurations at each field.
2. For the SFR (ZFR) protocol, texture configurations from the polarized (unpolarized) side of the full loop were used as the initializing texture configuration.
3. Next, for each H_{rev}, the field evolution of textures was simulated from H_{rev} to $+H_s$ (ZF) for SFR (ZFR) in steps of 2 mT.

4. For each $H_{\rm rev}$, the MAS spectrum for each individual field step (H) was simulated following the protocols used for manuscript Fig. 3 (see manuscript Experimental Section).
5. For each absorption spectrum, the peak positions of CF & KM resonance modes ($f_{\rm res}(H)$) were extracted by fitting to a normal Lorentzian function.
6. Following the protocols used for manuscript Fig.5 (manuscript Experimental Section), the dispersion of $f_{\rm res}(H)$ was plotted with respect to H/H_S for different $H_{\rm rev}$.
7. Likewise, domain (skyrmion) counting was also performed at each field step, giving $n_{\rm S}(H/H_{\rm S}; H_{\rm rev})$. The resulting resonance and texture plots are shown in Fig. S12. For ease of comparison, the field direction for ZFR has been flipped to be consistent with SFR.

The simulated results are overall consistent with the experiments (manuscript Fig. 5-6 and Fig. S10) for both SFR and ZFR protocols. Notably, for SFR, varying $H_{\rm rev}$ resulted in a non-linear modulation of the resonance dispersion, varying from linear to "W"-shaped over $0.5 < H/H_S < 0.8$ (Fig. S12(a)). For the "W"-shaped evolution (low $H_{\rm rev}$), texture evolution (Fig. S12(b)) shows increased skyrmion density due to stripe fission, followed by a drastic decrease due to their annihilation near $H_{\rm S}$.

Meanwhile, for ZFR, the resonance dispersion is linear for $0.7 < H/H_{\rm S} < 1$ (Fig. S12(c)). With varying $H_{\rm rev}$, the linear dispersion shows a systematic shift, corresponding to the associated shift of the skyrmion density plateau with $H_{\rm rev}$ (Fig. S12(d)). Overall, the trends are similar to experiments (manuscript Fig. 4-5). The simulations show larger offsets in skyrmion density across $H_{\rm rev}$ values, and a larger plateau over H, which can be attributed to their enhanced SNR and increased stability respectively, as compared to LTEM experiments.

Topological Charge. Considering a three-dimensional stack structure of chiral multilayers and their corresponding resonant features requires an understanding of topological charge across the stack. The complex hybrid domain wall chirality in chiral multilayer films arises due to the competition between the Dzyaloshinskii-Moriya interaction (DMI) and interlayer interactions[18,20]. A recent report accounts for the role of topological charge in a 20 repeated chiral multilayer, where large DMI competes with dipolar interactions, resulting in hybrid chirality across the stack structure[11].

Layer-Resolved Texture Character. For a realistic three-dimensional chiral multilayer stack, the domain wall (DW) chirality can exhibit nontrivial evolution across the stack, with "hybrid" character arising from the competition between DMI and interlayer dipolar and exchange interactions[18,20] and their corresponding resonant features requires an understanding of topological charge across the stack. A recent report on higher (20-) repeat multilayers shows that such hybrid chirality, manifesting as varying topological charge across the stack, can produce complex resonance characteristics[11]. To investigate the potential role of hybrid chirality in our work, we examine the layer-wise magnetization configuration of the 12 repeat Fe(0.3)/Co(0.7) multilayer. The simulations were performed for a single skyrmion (Fig. S13(a)) and employ an interlayer exchange coupling (IEC) of $H_{\rm IEC} = 250$ mT between layers, following detailed measurements of the non-magnetic spacer[21]. For our magnetic parameters, we observe columnar skyrmions with fixed chirality throughout the 12-repeat stack. This is verified by the layer-wise topological charge Q, defined as:

$$Q = \frac{1}{4\pi} \int m \cdot (\partial_x m \times \partial_y m)\, dr \tag{S7}$$

As shown in Fig. S13(b), Q is constant throughout the stack. In contrast to prior works[11,18], for our case, fixed chirality textures are stabilized by the interplay of strong DMI and finite IEC across moderate stack repetitions. Meanwhile, a slight, monotonic variation in DW width is observed across layers (reducing from bottom to top), likely due to interlayer dipolar interactions. These subtle variations are undetected by MAS spectra, underscore the robustness of the columnar textures in our work, with stable chirality enabling the observed resonance features.

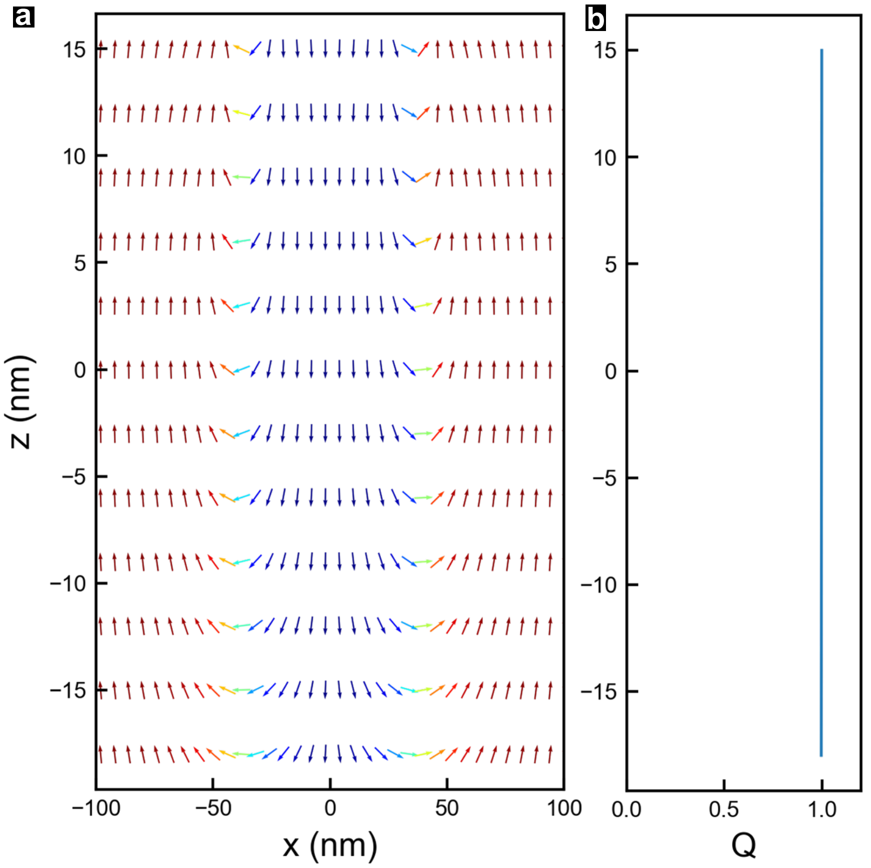

Figure S13. **Layer-wise Magnetization of a Skyrmion.** (a) Cross-sectional view of layer-wise magnetization configuration of a skyrmion across the 12-repeat Fe(0.3)/Co(0.7) stack. Arrows and color-scale represent the magnetization ($\mathbf{m}$) and its OP component (m_z), respectively. (b) Topological charge, Q calculated across each layer.

* anjan@nus.edu.sg

[1] S. Woo, K. Litzius, B. Krüger, M.-Y. Im, L. Caretta, K. Richter, M. Mann, A. Krone, R. M. Reeve, M. Weigand, P. Agrawal, I. Lemesh, M.-A. Mawass, P. Fischer, M. Kläui, G. S. D. Beach. Observation of room-temperature magnetic skyrmions and their current-driven dynamics in ultrathin metallic ferromagnets. *Nature Materials* **2016**, *15*, 5 501.

[2] T. Böttcher, T. S. Suraj, X. Chen, B. Sinha, H. R. Tan, H. K. Tan, R. Laskowski, B. Hillebrands, M. Kostylev, K. H. Khoo, A. Soumyanarayanan, P. Pirro. Quantifying symmetric exchange in ultrathin ferromagnetic films with chirality. *Phys. Rev. B* **2023**, *107* 094405.

[3] H. T. Nembach, T. J. Silva, J. M. Shaw, M. L. Schneider, M. J. Carey, S. Maat, J. R. Childress. Perpendicular Ferromagnetic Resonance Measurements of Damping and Landé g-Factor in Sputtered $(Co_2Mn)_{1-x}Ge_x$ Thin Films. *Phys. Rev. B* **2011**, *84* 054424.

[4] J. M. Shaw, H. T. Nembach, T. J. Silva. Determination of Spin Pumping as a Source of Linewidth in Sputtered $Co_{90}Fe_{10}$/Pd Multilayers by Use of Broadband Ferromagnetic Resonance Spectroscopy. *Phys. Rev. B* **2012**, *85* 054412.

[5] C. Kittel. Interpretation of Anomalous Larmor Frequencies in Ferromagnetic Resonance Experiment. *Physical Review* **1947**, *71*, 4 270.

[6] M. J. Benitez, A. Hrabec, A. P. Mihai, T. A. Moore, G. Burnell, D. McGrouther, C. H. Marrows, S. McVitie. Magnetic microscopy and topological stability of homochiral néel domain walls in a pt/co/alox trilayer. *Nature Communications* **2015**, *6*, 1 8957.

[7] X. Chen, M. Lin, J. F. Kong, H. R. Tan, A. K. C. Tan, S.-G. Je, H. K. Tan, K. H. Khoo, M.-Y. Im, A. Soumyanarayanan. Unveiling the emergent traits of chiral spin textures in magnetic multilayers. *Advanced Science* **2022**, *9*, 6 2103978.

[8] X. Chen, E. Chue, J. F. Kong, H. R. Tan, H. K. Tan, A. Soumyanarayanan. Thermal Evolution of Skyrmion Formation Mechanism in Chiral Multilayer Films. *Physical Review Applied* **2022**, *17*, 4 044039.

[9] A. K. C. Tan, J. Lourembam, X. Chen, P. Ho, H. K. Tan, A. Soumyanarayanan. Skyrmion generation from irreversible fission of stripes in chiral multilayer films. *Physical Review Materials* **2020**, *4*, 11 114419.

[10] H. Maier-Flaig, S. T. B. Goennenwein, R. Ohshima, M. Shiraishi, R. Gross, H. Huebl, M. Weiler. Note: Derivative divide, a method for the analysis of broadband ferromagnetic resonance in the frequency domain. *Review of Scientific Instruments* **2018**, *89*, 7 076101.

[11] T. Srivastava, Y. Sassi, F. Ajejas, A. Vecchiola, I. Ngouagnia Yemeli, H. Hurdequint, K. Bouzehouane, N. Reyren, V. Cros, T. Devolder, J.-V. Kim, G. De Loubens. Resonant dynamics of three-dimensional skyrmionic textures in thin film multilayers. *APL Materials* **2023**, *11*, 6 061110.

[12] L. Flacke, V. Ahrens, S. Mendisch, L. Körber, T. Böttcher, E. Meidinger, M. Yaqoob, M. Müller, L. Liensberger, A. Kákay, M. Becherer, P. Pirro, M. Althammer, S. Geprägs, H. Huebl, R. Gross, M. Weiler. Robust formation of nanoscale magnetic skyrmions in easy-plane anisotropy thin film multilayers with low damping. *Physical Review B* **2021**, *104*, 10 L100417.

[13] S. A. Montoya, S. Couture, J. J. Chess, J. C. T. Lee, N. Kent, M.-Y. Im, S. D. Kevan, P. Fischer, B. J. McMorran, S. Roy, V. Lomakin, E. E. Fullerton. Resonant properties of dipole skyrmions in amorphous Fe/Gd multilayers. *Physical Review B* **2017**, *95*, 22 224405.

[14] A. Soumyanarayanan, M. Raju, A. L. Gonzalez Oyarce, A. K. C. Tan, M.-Y. Im, A. P. Petrović, P. Ho, K. H. Khoo, M. Tran, C. K. Gan, F. Ernult, C. Panagopoulos. Tunable room-temperature magnetic skyrmions in Ir/Fe/Co/Pt multilayers. *Nature Materials* **2017**, *16*, 9 898.

[15] J. E. Davies, O. Hellwig, E. E. Fullerton, G. Denbeaux, J. B. Kortright, K. Liu. Magnetization reversal of Co/Pt multilayers: Microscopic origin of high-field magnetic irreversibility. *Physical Review B* **2004**, *70*, 22 224434.

[16] S. Chen, J. Lourembam, P. Ho, A. K. J. Toh, J. Huang, X. Chen, H. K. Tan, S. L. K. Yap, R. J. J. Lim, H. R. Tan, T. S. Suraj, M. I. Sim, Y. T. Toh, I. Lim, N. C. B. Lim, J. Zhou, H. J. Chung, S. T. Lim, A. Soumyanarayanan. All-electrical skyrmionic magnetic tunnel junction. *Nature* **2024**, *627*, 8004 522.

[17] A. Vansteenkiste, J. Leliaert, M. Dvornik, M. Helsen, F. Garcia-Sanchez, B. Van Waeyenberge. The design and verification of MuMax3. *AIP Advances* **2014**, *4*, 10 107133.

[18] W. Legrand, D. Maccariello, N. Reyren, K. Garcia, C. Moutafis, C. Moreau-Luchaire, S. Collin, K. Bouzehouane, V. Cros, A. Fert. Room-temperature current-induced generation and motion of sub-100 nm skyrmions. *Nano Letters* **2017**, *17*, 4 2703.

[19] S.-G. Je, D. Thian, X. Chen, L. Huang, D.-H. Jung, W. Chao, K.-S. Lee, J.-I. Hong, A. Soumyanarayanan, M.-Y. Im. Targeted writing and deleting of magnetic skyrmions in two-terminal nanowire devices. *Nano Letters* **2021**, *21*, 3 1253, pMID: 33481614.

[20] K. Fallon, S. McVitie, W. Legrand, F. Ajejas, D. Maccariello, S. Collin, V. Cros, N. Reyren. Quantitative imaging of hybrid chiral spin textures in magnetic multilayer systems by lorentz microscopy. *Phys. Rev. B* **2019**, *100* 214431.

[21] X. Chen, T. Tai, H. R. Tan, H. K. Tan, R. Lim, T. S. Suraj, P. Ho, A. Soumyanarayanan. Tailoring zero-field magnetic skyrmions in chiral multilayers by a duet of interlayer exchange couplings. *Advanced Functional Materials* **2024**, *34*, 1 2304560.
